# Proteolytic *Clostridium botulinum* (group I) – evaluation of predictive models for time-to-toxicity and identification of product characteristics that prevent growth and toxin formation in different types of foods

**DOI:** 10.3389/fmicb.2026.1898380

**Published:** 2026-09-03

**Authors:** Edgar Remmet Snoeck, Ioulia Koukou, Paw Dalgaard

**Affiliations:** Food Microbiology and Hygiene, DTU National Food Institute, Technical University of Denmark (DTU), Kgs. Lyngby, Denmark

**Keywords:** foodborne botulism, meat, vegetables, cheeses, model validation, safety by design

## Abstract

Preventing toxin formation by proteolytic *Clostridium botulinum* is critical for food safety but remains a challenge for example when developing or reformulating various products including sodium-reduced or nitrite-free foods. Validated predictive food microbiology models can support this process by identifying combinations of product characteristics and storage conditions that prevent growth and toxin formation. An existing cardinal parameter growth and growth boundary model (Koukou et al., 2022b), including the effect of 11 environmental factors and their interactions, was extended to predict time-to-toxicity (TTT) for proteolytic *C. botulinum*. Data from 962 published TTT experiments were compiled to evaluate performance for different versions of this model. Product characteristics, storage conditions and TTT were collected for meat, poultry, vegetables, seafood, dough, processed cheese, other cheeses and uncategorized foods. These data, with 648 TTT positive and 314 TTT negative, covered a wide range of environmental factors and included responses for 138 proteolytic *C. botulinum* strains. Model performance was evaluated using percentages of fail-dangerous, fail-safe and correct predictions along with bias and accuracy factors for predicted and observed TTT. Model performance was markedly different for non-dairy and dairy foods. For non-dairy foods and within a wide range of applicability, including 494 TTT-responses, the new and extensive Koukou-TTT-model had 0.2% fail-dangerous TTT predictions and bias/accuracy factors of 0.53/2.5. For dairy products (*n* = 312), including processed and other cheeses, the Koukou-TTT-model provided 26.0% fail-dangerous TTT predictions. This unacceptable model performance was primarily due to calcium complexation with citric and lactic acids which reduced the inhibiting effect of these organic acids in the studied dairy foods. The Koukou-TTT-model was modified to predict the effect of total, rather than undissociated, concentrations of citric and lactic acids in dairy foods. This modified model predicted 144 TTT-responses with 0.0% fail-dangerous TTT predictions and bias/accuracy factors of 0.37/4.42. The original and the modified growth and growth boundary Koukou-models can be used, within defined and wide ranges of applicability, to determine combinations of product characteristics and storage conditions where growth and TTT for proteolytic *C. botulinum* are prevented in meat, poultry, vegetables, seafood, dough and dairy products.

## 1 Introduction

Mesophilic proteolytic *Clostridium botulinum* (Group I) are important spore-forming pathogenic bacteria capable of growth in foods stored above 10–12 °C. Under favorable conditions, they can produce highly potent botulinum neurotoxins (BoNTs) primarily of types A, B and F. Food-borne botulism arises when spores germinate, proliferate, and generate BoNTs in foods that are consumed with no or little heat treatment. This disease is a serious neuroparalytic intoxication characterized by prolonged recovery and fatality rates of 5–10% (Anderson et al., 2011; Fleck-Derderian et al., 2018; Rawson et al., 2023). Spores of proteolytic *C. botulinum* are common in soil and may be present in various food raw materials and products as they have high resistance to heat and other food processing operations. Reported prevalence and concentrations of proteolytic *C. botulinum* spores vary widely between geographical regions and types of foods. One to two proteolytic spores/g have been reported for mushrooms and vegetarian sausages but much lower concentrations of below 0.0001 to about 0.01 proteolytic spores/g were detected for meat, seafood and dairy products (Dodds, 1994; Anderson et al., 2011; Lindström et al., 2010; Pernu et al., 2020). Nevertheless, botulism due to proteolytic *C. botulinum* has been caused worldwide by various types of both home-made and commercially produced vegetables (e.g., bottled or canned asparagus, bamboo shoots, chilli sauce, carrots, carrot juice, green beans; garlic in oil; green olive paste; hummus; olives; potato salad and soup; pruno; fermented/stinky tofu and tempeh), meat (e.g., cooked ham/pork, dried beef, meat roll, sausage, smoked ribs), poultry (e.g., chicken enchiladas), dairy (e.g., brie, cheese sauce, cheese spread, cottage cheese, mascarpone/tiramisu and nacho cheese) and seafood (e.g., clam chowder and fish soup) (Carter and Peck, 2014; Fleck-Derderian et al., 2018; Lúquez et al., 2021; Chaidoutis et al., 2022; Li et al., 2022; Franciosa, 2020; Advisory Committee on the Microbiological Safety of Food (ACMSF), 2023).

Proteolytic *C. botulinum* has been extensively studied and guidelines to manage their growth and toxicity through selected intrinsic product characteristics or heat treatment are well established. Foods at above 10 °C, should have a pH below 4.6, over 10% water phase salt (WPS), or undergo heat treatment equivalent to 121 °C for 3 min in sealed packaging (Anderson et al., 2011; Advisory Committee on the Microbiological Safety of Food (ACMSF), 2023). These controls, however, can be challenging for many foods where costs, sensory and technological characteristics are negatively influenced. It is therefore useful to apply combinations of preserving factors that efficiently prevent growth and toxin formation while at the same time have little or no negative effect on sensory and technological characteristics. To identify such combinations experimentally can be laborious and costly. Validated predictive food microbiology models may facilitate this task as they have previously been used to support formulation of recipes that prevented growth of different food-borne pathogens (Ter Steeg and Cuppers, 1995; Dalgaard and Mejlholm, 2019; Koukou et al., 2021; Koukou et al., 2022a; Maktabdar et al., 2025a, 2025b). This approach is most relevant for proteolytic *C. botulinum* and different models including the effect of several environmental factors have been suggested to predict their growth, growth boundary or probability of growth/toxin formation (Roberts et al., 1981, 1982; Robinson et al., 1982; Gibson et al., 1987; Dodds, 1989; Whiting and Call, 1993; Whiting and Strobaugh, 1998; Zhao et al., 2002; Koukou et al., 2022b). Limited data is available for growth responses of *C. botulinum* group I in various foods, and this is caused by challenges with access to relevant strains, laboratory requirements and lack of specific enumeration methods. As a result, the evaluation and validation of models to predict growth remains very little studied even for simple models including exclusively the effect of storage temperature (Juneja and Marks, 1999; Juneja et al., 2021a, 2021b; Juneja et al., 2022; Koukou et al., 2022b). Much more time-to-toxicity (TTT) data exist for proteolytic *C. botulinum* in various foods and several models are available to predict the combined effect of product properties on TTT in processed cheese and some other foods (Ivey and Robach, 1978; Tanaka et al., 1986; Ter Steeg and Cuppers, 1995; Schaffner et al., 1998; Glass et al., 2017). Importantly, growth and toxin formation models have not been evaluated and validated for many of the types of foods where management of proteolytic *C. botulinum* is relevant. It is therefore interesting to evaluate the ability of models to predict TTT data for various types of foods, so that validated models can potentially be used to facilitate formulation of recipes for products where growth and toxin-formation is prevented. Procedures to evaluate and validate the performance of growth and growth boundary models are available and these may be adapted for TTT models. In brief, these procedures include (i) collection of relevant product characteristics, storage conditions and growth or TTT responses, (ii) calculation of indices for model performance and (iii) determination of a model’s range of applicability (RoA) within which it has been successfully validated with respect to relevant factors such as food matrix, product characteristics and storage conditions (Ross, 1996; Mejlholm et al., 2010; Koukou et al., 2022a).

The objective of the present study was to evaluate performance of the previously developed Koukou et al. (2022b) growth and growth boundary model by using available TTT data and to determine conditions under which this model can predict if growth and toxin formation is prevented. This included (i) collect available TTT data for proteolytic *C. botulinum* and corresponding product characteristics and storage temperatures from the scientific literature, (ii) expand the extensive growth and growth boundary model of Koukou et al. (2022b) to predict TTT as the time required for a defined log-increase in cell concentrations, (iii) use the collected TTT data to evaluate and determine RoA for the new Koukou et al. (2022b) TTT-model and (iv) evaluate effects of initial spore concentrations, type of food matrices, contents of nitrite, calcium and lipid as well as ranges of environmental factors on model performance.

## 2 Materials and methods

### 2.1 Extraction of TTT data and product characteristics from scientific literature for model evaluation

TTT data and product characteristics were extracted from the scientific literature to evaluate performance and potentially validated the cardinal parameter growth and growth boundary model previously developed by Koukou et al. (2022b). Thus, the collected TTT data was not used to refit cardinal parameter values for the 11 environmental factors included in this model. This study did not include a systematic review. Instead, the DTU Findit digital library^1^ was queried for peer-reviewed publications and their respective references in which TTT challenge tests for proteolytic *C. botulinum* of BoNT type A, B or F were reported. Data not studied as part of the present model evaluation included (i) TTT data determined using liquid laboratory media, (ii) TTT data determined in studies where key preserving factors could not be estimated, (iii) data for probability of toxin formation and (iv) data used to estimate parameter values in the new Koukou et al. (2022b) TTT model (Section 2.2).

Validation data included 962 TTT responses for meat products, poultry products, seafoods, dough products, vegetables, and uncategorized foods as well as for processed and other cheeses. Initial concentrations of proteolytic *C. botulinum* spores (CFU/g), last-time-negative (LTN) and first-time-positive (FTP) for TTT (days), storage time (days), and relevant environmental factors including temperature, pH, water phase salt/NaCl (WPS) or water activity (a_w_), concentrations of moisture or dry matter (DM), lipid, organic acids, phosphate ions, nitrite and calcium were collected. When information on relevant factors was not reported, missing values were, if possible, estimated from similar products as described by Mejlholm et al. (2010) and Koukou et al. (2022a) (Supplementary Tables S1, S2). Otherwise, TTT data were excluded from the present validation study. Specifically, values for pH, when not reported, were obtained from the Center for Food Safety and Applied Nutrition food composition references [U.S. FDA CFSAN (United States Food and Drug Administration, Center for Food Safety and Applied Nutrition), 2000, 2007] and from Kim et al. (2016). Values for lactic acid were retrieved from Dando (1969), Karahadian and Lindsay (1987), Matsumoto and Yamanaka (1990), Smith et al. (1992), Puolanne et al. (2002), Shumilina et al. (2015), Jun et al. (2017), Zybert et al. (2020) and Martinez-Rios et al. (2020). Values for acetic acid in pickled cucumbers were retrieved from Lu et al. (2002). Benzoic acid and lipid concentrations in commercial caviars were retrieved from Vasconi et al. (2020). Values for WPS, DM, and lipid in lotus root were retrieved from Showkat et al. (2021). Concentrations of ortho-phosphate (P1), pyro-phosphate (P2) and tri-phosphate (P3) in commercial phosphate preparations were obtained from Martinez-Rios et al. (2019). If not directly reported, then water phase concentrations of NaCl, organic acids and phosphate ions were calculated as previously described (Ross and Dalgaard, 2004). To calculate the concentrations of water phase benzoic and sorbic acids, the partitioning between lipid and water phases in products were considered (Equation 1; Koukou et al., 2022b; Maktabdar et al., 2025b).

$$OA_{\mathrm{wp}}=\frac{OA_{p}.100}{\left[1+K_{p}(\frac{\theta }{1-\theta })]\cdot (100-\mathrm{DM}+\mathrm{Fat}\right)}\;$$ (1)

Where *OA_wp_* is the concentration of benzoic or sorbic acids in water phase (ppm), *OA_p_* is the total concentration of each of these organic acids in the product (ppm), *DM* is the dry matter concentration (%), and *Fat* is fat content (%). *θ* is the fraction of fat in the water and fat phase, and *K_p_* is the partition coefficient between water and fat which was set to 7.22 and 4.19 for benzoic and sorbic acid, respectively.

### 2.2 Increase in cell concentration corresponding to toxin formation and prediction of TTT

The growth model by Koukou et al. (2022b) was expanded to predict TTT and this was obtained by determination of the increase in cell concentrations corresponding to toxin formation (*ΔN_TTT_*), an approach previously used in other studies (Ter Steeg and Cuppers, 1995; Koukou et al., 2021). *ΔN_TTT_* was determined using 15 kinetics extracted from the scientific literature and where both growth (log CFU/g) and toxin formation by proteolytic *C. botulinum* were reported over time (Table 1). These data were not part of the 962 TTT observations used for model evaluation. Observed TTT responses were, for each challenge test, determined as the average of LTN and FTP (Section 2.1) and the corresponding cell concentration, log(*N_TTT_*, CFU/g), was obtained by interpolation. *ΔN_TTT_* was calculated as previously suggested for non-proteolytic *C. botulinum* (Equation 2; Koukou et al., 2021).

$$\Delta N_{\mathrm{TTT}}=10^{\frac{\sum (\log(N_{\mathrm{TTT}})-\log(N_{0}))}{n}}$$ (2)

Where *N_0_* (CFU/g) is the initial inoculum concentration in each of the 15 experiments (Table 1).

**Table 1:** Increase in cell concentrations of proteolytic *C. botulinum* at the time of toxin formation (ΔN_TTT_).

Code	Product	Storage temp. (°C)	a_w_	pH	Log (N_0_, CFU/g) ^b^	log (N_TTT_, CFU/g) ^c^	Log (N_max_, CFU/g) ^d^	Log (ΔN_TTT_, CFU/g) ^e^	References
D1	3.5% fat milk	18	0.999 ^a^	6.3	3.6	5.9	6.6	2.2	Kaufmann and Marshall (1965)
D2	3.5% fat milk	18	0.999 ^a^	6.3	3.3	6.4	6.7	3.1	
D3	3.5% fat milk	18	0.999 ^a^	6.3	3.3	4.8	6.5	1.5	
D4	Parma ham (shank)	20	0.975	5.8	0.8	4.1	5.5	3.3	Merialdi et al. (2016)
D5	Parma ham (shank)	20	0.970	6.3	0.8	4.2	5.4	3.4	
D6	TYG^f^	30	0.996	7.3	3.6	5.7	8.0	2.1	Briozzo et al. (1983)
D7	TYG^f^ with cheese whey powder	30	0.972	5.6	3.7	6.0	8.3	2.3	
D8	TYG^f^ with cheese whey powder	30	0.965	5.7	3.7	5.0	6.7	1.3	
D9	TYG^f^ with cheese whey powder	30	0.965	5.7	3.7	4.5	6.5	0.8	
D10	English style crumpets	25	0.990	6.5	2.7	5.0	6.3	2.3	Daifas et al. (1999b)
D11	English style crumpets	25	0.990	6.5	2.7	4.6	5.8	1.9	
D12	English style crumpets	25	0.990	6.5	2.7	4.1	6.3	1.4	
D13	English style crumpets	25	0.990	6.5	2.7	5.2	6.7	2.5	
D14	English style crumpets	25	0.990	6.5	2.7	3.7	5.3	1.0	
D15	English style crumpets	25	0.990	6.5	2.7	5.2	6.2	2.5	
D1-D15 (avg ± SD)								2.11 ± 0.79	

a Estimated from similar products as described by Koukou et al. (2022a) and Mejlholm et al. (2010).

b Initial spore concentration (*N*_0_).

c Geometric average of population densities at the last time negative and first time positive toxicity observations.

d Maximum population density (N_max_).

e Growth-to-toxicity; log (ΔΝ_TTT_) = log(N_TTT_) – log(N_0_).

f Modified trypticase-yeast extract-glucose medium with 5% (w/w) added cysteine.

TTT was predicted using Equation 3 as previously suggested by Koukou et al. (2021).

$$\begin{array}{l} \text{Predicted}\;\mathrm{TTT}\;(d)=t_{\mathrm{lag}}+\text{time for}\;\Delta N_{\mathrm{TTT}}\;\text{growth}\\ =\frac{\mathrm{RLT}\cdot \mathrm{Ln}(2)}{\mu_{\max}\;(h)\cdot 24}+\frac{\ln(\frac{N_{\max}}{N_{0}}-1)-\ln(\frac{N_{\max}}{N_{0}\cdot \Delta N_{\mathrm{TTT}}}-1)}{\mu_{\max}(h)\cdot 24} \end{array}$$ (3)

Where predicted TTT is the time in days required for growth from the initial cell concentration log (*N_0_*, CFU/g) to log(*N_0_*, CFU/g) + log(*ΔN_TTT_*, CFU/g) with maximum specific growth rate (*μ_max_*, 1/h), relative lag time (*RLT*) and maximum population density (*N_max_*, CFU/g) determined by the model of Koukou et al. (2022b). This expanded Koukou et al. (2022b) model for TTT prediction used values of 24.0 for *RLT* and 6.8 log CFU/g for log(*N_max_*, CFU/g) as determined from literature growth data for proteolytic *C. botulinum* (Koukou et al., 2022b). The model of Koukou et al. (2022b) was previously developed as an extensive growth and growth boundary model for a cocktail of spores for three *C. sporogenes* strains. This cardinal parameter model includes, a reference growth rate (*μ_ref_*) of 2.50 h^−1^ at 37 °C and accounts for the growth inhibiting effect of 11 environmental factors: temperature, pH, WPS, acetic (AAC), benzoic (BAC), citric (CAC), lactic (LAC) and sorbic (SAC) acids and three phosphate melting salts being ortho- (P1), pyro- (P2) and tri-phosphate (P3). It also incorporates the interactive effects of these factors on *μ_max_*. Terms in this model included water activity (a_w_) as calculated from % WPS and concentrations of undissociated organic acids as calculated from water phase concentrations of individual acids and pH. The term for the ortho-phosphate ion in the water phase included a value of 0.83% below which no growth inhibiting effect of this melting salt was predicted (Koukou et al., 2022b). The growth boundary and conditions that prevent growth were predicted from the combined inhibitory effect of individual environmental factors and of interaction between these factors. Terms for each environmental factor had a value between zero and one corresponding, respectively, to no growth and no inhibition of growth. Each term included a cardinal parameter value, for example, the minimum inhibitory concentration (MIC-value) of an undissociated organic acids in water phase or of ions of the three phosphate melting salts. The effect of interaction between the model’s 11 environmental factors was expressed as a *ψ*-value, with ψ < 0.5 for no growth inhibiting effect of interaction, 0.5 < ψ < 1.0 for some but not a complete growth inhibiting effect of interaction and ψ ≥ 1.0 for growth being prevented due to the effect of interaction between environmental factors (Le Marc et al., 2002; Koukou et al., 2022b).

### 2.3 Evaluation of TTT-model for non-dairy products

Performance of the Koukou et al. (2022b) TTT model (Section 2.2) was evaluated using data collected for meat products, poultry products, seafoods, dough products, vegetables, and uncategorized foods (Section 2.1; Table 2). To compare collected and predicted TTT responses the percentage of fail-dangerous, fail-safe and correct TTT predictions were calculated as described by Koukou et al. (2021, 2022a). Specifically, (i) fail-dangerous TTT predictions were instances where TTT was experimentally observed but not predicted (*μ_max_* = 0; ψ ≥ 1), (ii) fail-safe TTT predictions were instances where TTT was not observed experimentally but predicted (*μ_max_* > 0; ψ < 1), and (iii) correct TTT predictions were instances where TTT was both experimentally observed and predicted (*μ_max_* > 0; ψ < 1). Precise models should provide 0% fail-dangerous, 0% fail-safe and 100% correct predictions but due to variability, for example, in product characteristics, a small percentage of fail-safe and fail-dangerous predictions can be observed, even for precise models. Previously, validation studies found acceptable *Listeria monocytogenes* growth models to have >75% correct, <15% fail-safe and <10% fail-dangerous predictions (Martinez-Rios et al., 2020; Mejlholm et al., 2010). However, fail-dangerous TTT predictions were exclusively accepted within the range of applicability when their ψ-values were close to the growth boundary (ψ = 1.0) and well below the ψ-value limit of 2.0 used to design products that efficiently prevent growth and toxin-formation by *C. botulinum* (Koukou et al., 2021; Koukou et al., 2022a).

**Table 2:** Observed and predicted time-to-toxicity (TTT) for proteolytic *C. botulinum* in non-dairy food and overview of challenge tests data including product characteristics as extracted from literature.

Product/category	NTO/*n* ^a^	*N*_0_ ^b^ (log CFU/g)	Temp. (°C)	WPS ^c^ (%)		a_w_ ^d^	pH	Organic acids in water phase (mg/L) ^g^			Organic acids in product (mg/kg) ^g^		Phosphate ions in water phase (%) ^g^		Time-to-toxicity (days)		References
								AAC	CAC	LAC	BAC	SAC	P2	P3	Observed^h^	Predicted	
Meat products (*n* = 233)																	
Smoked bacon, 0–340 ppm NaNO₂	26/48	1.6–3.6	7.0–27.0	5.0		0.971	6.40 ᵉ	0	0	2,000 ᵉ	0	0	0.00	0.00	4–70	4 or NTP ^i^	Christiansen et al. (1974)
Pork liver sausage, 0–150 ppm NaNO₂	5/47	1.9–4.9	27.0	3.8-5.4		0.968–0.979	6.00–6.10 ᵉ	0	0	2,000 ᵉ	0	0	0.00	0.00	4–25	2–3 or NTP ^i^	Hauschild et al. (1982)
Ham and bacon, 0–120 ppm NaNO₂	1/24	2.6	30.0	5.6-5.9 ᵉ		0.965–0.966	5.65–6.40	0–2,399	0–3,189	1,000–4,752	0	0–1,000	0.00	0.00	4–21	4–52 or NTP ^i^	Huhtanen et al. (1983)
Bacon, 0–120 ppm NaNO₂	2/8	3.0	27.0	3.1–4.3 ᵉ		0.975–0.982	6.10–6.35	0	0	2,000 ^e^	0	117–2,105	0.00–0.21	0.00–0.59	19–39	2–4	Ivey et al. (1978)
Fresh pork	1/3	4.0	15.0–25.0	0.7 ᵉ		0.996	5.70	0	0	5,000 ᵉ	0	0	0.00	0.00	2–14	2–12	Lambert et al. (1991)
Pulled pork	0/1	3.0	12.8	3.2		0.982	5.91	0	0	4,473^e^	0	0	0.00	0.00	7	37	Glass et al. (2024)
Pork slurry, 40 ppm NaNO_2_	24/96	−0.4 − 1.6	15.0–35.0	2.0–4.2		0.976–0.989	5.70–6.56	0	0	2,272^e^	0	0–1,941 ᵉ	0.00	0.00	-^f^	-^f^	Roberts et al. (1982)
Seasoned seared beef sirloin	3/6	3.5	16.0–30.0	1.0		0.995	5.80	0	0	6,452–68,663^e^	0	0	0.00	0.00	1–8	2–9 or NTP ^i^	Meng and Genigeorgis (1994)
Poultry products (*n* = 77)																	
Turkey frankfurter, 150 ppm NaNO₂	9/30	2.8–3.1	27.0	1.4–4.2		0.976–0.992	6.17–6.58	0	0	2,000 ᵉ	0	0	0.00–0.25	0.00–0.41	2–6	1–3	Barbut et al. (1986)
Cook in bag ground turkey breast	0/5	2.7	27.0	2.0–2.1		0.988–0.989	6.29–6.34	0	0	0–41,314	0	0	0.00	0.00	3–7	1–3	Maas et al. (1989)
Ground turkey breast	3/14	2.7	28.0	2.0		0.989	5.50–6.50	0–64,364	0–65,459	0–70,339	0	0	0.26	0.00	1–16	1–9 or NTP ^i^	Miller et al. (1993)
Chicken frankfurter, 40 ppm NaNO_2_	1/19	2.7	27.0	3.3		0.981	5.96–6.19	0	0	2,000 ᵉ	0	0–2,000	0.00–0.43	0.00–0.51	3–27	2–6	Nelson et al. (1983)
Chicken a-la king	0/2	1.3–3.3	30.0	0.7		0.996	6.02–6.08	0	0	2,000 ᵉ	0	0	0.00	0.00	1	1	Saleh and Ordal (1955)
Seasoned grilled chicken breast	2/6	3.5	16.0–30.0	0.5		0.997	5.90	0	0	6,359–52,350 ᵉ	0	0	0.00	0.00	2–22	1–7 or NTP ^i^	Meng and Genigeorgis (1994)
Pulled turkey	0/1	3.0	12.8	1.5		0.983	6.08	0	0	2,997	0	0	0.00	0.00	7	24	Glass et al. (2024)
Seafoods (*n* = 107)																	
Brined cold and warm water shrimp	5/6	2.0	8.0–25.0	2.3–3.3	0.981–0.987	5.70–5.90	0	3,588–4,100	0	735–767	632–768	0.00	0.00	4	NTP^i^	Dalgaard and Jørgensen (2000)	
Black lumpfish caviar	35/52	2.5–2.8	30.0	2.3–7.1	0.957–0.987	5.00–5.80	0	1,921^e^	0	840^e^	0	0.00	0.00	21	2,276 or NTP^i^	Hauschild and Hilsheimer (1979)	
Blended autoclaved mullet and shrimp	0/8	4.1–4.9	15.0–30.0	0.3^e^	0.999	6.50–7.00	0	0	500–2,000^e^	0	0	0.00	0.00	1–6	1–6 or toxicity at N_TTT_ > Nma_x_^j^	Lalitha and Gopakumar (2001)	
Poached minced salmon	2/6	3.5	16.0–30.0	0.3	0.998	6.40	0	0	5,714–60,816^e^	0	0	0.00	0.00	1–14	1–8 or NTP^i^	Meng and Genigeorgis (1994)	
Hot smoked salmon	4/25	2.0–4.0	25.0	2.3–9.5	0.941–0.988	6.10	0	0	7,000^e^	0	0	0.00	0.00	4–18	2–522 or NTP^i^	Pelroy et al. (1982)	
Salmon fillets	0/2	1.8	22.2	0.1^e^	0.999	6.30^e^	0	0	5,000^e^	0	0	0.00	0.00	2	2	Stier et al. (1981)	
Crabmeat	6/8	4.3	4.0–26.0	2.1^e^	0.988	6.30	0	0	1,000^e^	0	0	0.00	0.00	2–3	2–1,325 or NTP^i^	Solomon et al. (1977)	
Dough products (*n* = 34)																	
English style crumpets	0/4	2.7	25.0	2.6^e^	0.985	6.50–8.30	0	0	0	0	0	0.00	0.00	4–7	1–2	Daifas et al. (1999a)	
Crumpets, pizza crust, and bagels	3/9	4.7	25.0	1.0–2.6^e^	0.985–0.994	5.63–6.00	0	0	0	0	0	0.00	0.00	21	NTP^i^ or toxicity at N_TTT_ > N_max_ ^j^	Daifas et al. (1999b)	
Meat- and cheese tortellini, plain- and spinach fettucini, and plain linguine	6/9	2.0	30.0	0.0–0.5^e^	0.997–1.000	5.70–6.90	0	0	0–1,000^e^	0	0	0.00	0.00	4–39	1	Glass and Doyle (1991)	
Spaghetti in meat sauce	4/12	3.0	15.0	0.0–4.0	0.977–1.000	4.40–6.00	0	0	2,000^e^	0	0	0.00	0.00	7–39	7–217 or NTP^i^	Simpson et al. (1995)	
Vegetables (*n* = 149)																	
Onion, butternut squash, rutabaga, romaine lettuce, coleslaw mix, stir-fry mix, salad mix	10/19	1.0–3.0	15.0–25.0	0.2–0.3^e^	0.998–0.999	5.20–6.00^e^	0	0	0	0	0	0.00	0.00	2–14	1–72	Austin et al. (1998)	
Cooked mashed potatoes	3/9	5.0	25.0	0.0–5.9	0.965–1.000	4.83–6.10	0	0	0	0	0	0.00	0.00	5–25	NTP^i^ or toxicity at N_TTT_ > N_max_ ^j^	Dodds (1989)	
Raw, cooked, and autoclaved lotus roots	1/4	4.0	30.0	0.0 ^e^	1.000	5.85	0	0	0	0	0	0.00	0.00	3	1	Hayashi et al. (1986)	
Cucumber puree	7/18	2.0–6.0	30.0	1.0	0.994	4.50–6.20	256–1,061 ^e^	0	0	0	0	0.00	0.00	4–18	NTP^i^ or 1–6	Ito et al. (1976)	
Chopped coleslaw and chopped carrots, broccoli, sliced carrots, green beans, shredded lettuce	13/15	2.0	4.0–21.0	0.0 ^e^	1.000	6.23–6.75	0	0	0	0	0	0.00	0.00	6–9	NTP^i^ or 2–14	Larson et al. (1997)	
Pasteurized potato	5/20	1.0–5.0	25.0–30.0	0.0 ^e^	1.000	5.70 ^e^	0	0	0	0	0	0.00	0.00	3–8	1–2 or toxicity at N_TTT_ > N_max_ ^j^	Lund et al. (1988)	
Pea soup	2/6	3.0	15.0–35.0	2.1 ^e^	0.989	6.20	0	0	0	0	0	0.00	0.00	1–5	1–8	Skinner et al. (1999)	
Sauteed Spanish and yellow onions	1/30	0.0–4.2	35.0	0.0	1.000	5.40	0	0	0	0	0	0.00	0.00	1–4	1	Solomon and Kautter (1986)	
Raw peeled potato slices	4/8	2.7–4.1	22.0	0.0 ^e^	1.000	5.70 ^e^	0	0	0	0	0	0.00	0.00	3	2	Solomon et al. (1994)	
Baked Russet Burkbank potatoes	0/20	1.0–5.0	22.0–30.0	0.0 ^e^	1.000	5.70 ^e^	0	0	0	0	0	0.00	0.00	3–7	1–2 or toxicity at N_TTT_ > N_max_ ^j^	Sugiyama et al. (1981)	
Uncategorized foods (*n* = 50)																	
Raw and hard boiled eggs	3/16	1.0–4.0	30.0	0.5 ^e^	0.997	8.00–8.50	0	0	0	0	0	0.00	0.00	3–7	1	Lubin et al. (1985)	
Fig purees	10/16	2.0–6.0	30.0	0.0 ^e^	1.000	5.10–5.70	0	0	0	0	0	0.00	0.00	7–23	1–9 or toxicity at N_TTT_ > N_max_ ^j^	Ito et al. (1978)	
Steamed rice with and without amylase or Bacillus subtilis	1/8	1.0	30.0	0.0	1.000	6.50–6.70	0	0	0	0	0	0.00	0.00	1–8	1	Kasai et al. (2005)	
Steamed rice	2/10	2.0	30.0	0.0	1.000	4.70- 6.80	0	0	0	0	0	0.00	0.00	7–126	NTP^i^ or 1–4	Kimura et al. (2008)	

a Number of no toxicity observed responses (NTO) and total number of TTT responses (*n*).

b Initial spore concentration (*N*_0_).

c Water phase NaCl (WPS).

d The water activity (a_w_) calculated from WPS using: a_w_ = 1–0.0052471 * WPS − 0.00012206 * WPS^2^ (Ross and Dalgaard, 2004).

e Estimated from product characteristics or similar products, as described by Koukou et al. (2022a) and Mejlholm et al. (2010).

f Probability of toxin formation was reported only after 180 days. Therefore, these data were used only for qualitative evaluation of toxicity/no-toxicity predictions.

g Acetic acid (AAC), benzoic acid (BAC), citric acid (CAC), lactic acid (LAC), sorbic acid (SAC), ortho-phosphate (P1), pyro-phosphate (P2), and tri-phosphate (P3).

h Average of last-time-negative and first-time-positive toxicity observations transformed to days where toxicity was observed.

i No toxicity predicted (NTP).

j Population density at toxicity (N_TTT_) and maximum population density (N_max_).

Additionally, bias (B_f-TTT_) and accuracy (A_f-TTT_) factors for predicted and observed TTT were calculated exclusively when both the observed and predicted TTT responses were available as numeric values (Koukou et al., 2021, 2022a). For B_f-TTT_ the performance limits suggested by Ross (1999) for generation times were used, as previously applied by Koukou et al. (2021, 2022a) in TTT model evaluations. B_f-TTT_ values between 0.9 and 1.05 indicate good model performance, B_f-TTT_ values of 1.06–1.15 or 0.7–0.9 indicate acceptable model performance and B_f-TTT_ values <0.7 or >1.15 were considered unacceptable. A_f_–values in evaluation studies will increase from 1.0 by 0.10–0.15 for every environmental factor used in a model (Ross et al., 2000). The evaluated TTT model predicted the effects of up to seven environmental factors for specific TTT responses, and acceptable A_f-TTT_–values should therefore be below 1.7–2.1.

To evaluate potential major effects of food matrices and of initial spore concentrations on TTT in challenge tests, the 962 observed and collected TTT responses were divided into non-dairy (*n* = 650) and dairy (*n* = 312) products. Within each product category observed and predicted TTT responses were compared depending on ranges of the initial spore concentrations. Subsequently, observed and predicted TTT responses were compared for sub-groups of non-dairy products. Meat- and poultry-products included treatments with and without added nitrite (Table 2) and these responses were evaluated both together and separately. Predictions by the Koukou et al. (2022b) TTT model were performed (i) for the full range of product characteristics and storage temperatures relevant to the product sub-groups studied, (ii) within the original RoA of the Koukou et al. (2022b) model as determined from growth responses and (iii) within an optimized RoA, determined as described in section 2.5.

### 2.4 Evaluation of TTT-models for dairy products

TTT responses for dairy products (*n* = 312), including processed cheeses (*n* = 256) and other cheeses (*n* = 56), were compared with predicted TTT-values as described above (Section 2.3). However, due to the calcium content of dairy products and its binding to CAC and LAC, modifications of the Koukou et al. (2022b) TTT model were also evaluated.

#### 2.4.1 Correction for binding of citric and lactic acids by calcium

Dairy products can contain notable concentrations of calcium (Ca), for example, from 1,200 to 1,800 mg/kg of total Ca in milk and yoghurt, whereas some cheeses can contain up to 10,000 mg/kg (Shkembi and Huppertz, 2022; Guinee et al., 2002; Guinee and O’Kennedy, 2009; Deshwal et al., 2024; RIVM, 2025). Ca in the water phase is known to form complexes with CAC and LAC and thereby lower the concentration of free undissociated acids and their growth inhibitory effects (Muller et al., 1998; Graham and Lund, 1986; Wood et al., 1975; Walstra et al., 2005). Accordingly, the present study accounted for Ca complexation when estimating the concentrations of free CAC and LAC in dairy products and this procedure comprised three steps. Firstly, the Ca concentration in the aqueous phase of each dairy product was estimated from the total Ca content. Secondly, the partitioning of CAC, and LAC among free, dissociated, undissociated, and Ca-complexed fractions was calculated from a system of equilibrium and mass-balance equations (Equations 3–7). Thirdly, the calculated concentrations of free CAC and LAC were used as inputs for evaluation of the Koukou et al. (2022b) TTT model.

For cheeses studied by Ter Steeg and Cuppers (1995), Glass and Johnson (2004a), Tanaka et al. (1979), Wagenaar and Dack (1958a, 1958b, 1958c), and Ter Steeg et al. (1995), total Ca concentrations were estimated from the reported formulations using ingredient specifications (U.S. Dairy Export Council, 2001) or, where necessary, from the NEVO food composition database (RIVM, 2025) and Karahadian and Lindsay (1987). The resulting total Ca concentrations ranged from 2,505 to 4,934 mg/kg. For the processed cheeses studied by Glass et al. (2017) and Tanaka (1982), complete recipes were not available. Therefore, a total Ca concentration of 4,820 mg/kg was assumed, corresponding to the arithmetic mean of the processed-cheese values reported by Guinee and O’Kennedy (2009). Total Ca concentrations in the studied dairy products were reduced by 26.7% to pragmatically account for binding to compounds in the solid colloidal phase (Pereira et al., 2019). Calculated water phase Ca concentrations are presented in Supplementary Table S2.

The measured or estimated total water phase concentrations of Ca, CAC, LAC, and pH were used to determine concentrations of bound or complexed Ca and dissociated or undissociated concentrations of CAC or LAC. These concentrations were estimated by a set of simultaneous competing equilibria (Equations 3, 4), governed by Ca complexation, acid dissociation equilibrium constants and the overall Ca, CAC, and LAC mass balances (Equations 5, 6, 7).

$$\begin{array}{l} \begin{array}{c} \;\;\\ H_{3}\mathrm{CAC}+Ca^{2+} \end{array}\;\;\begin{array}{c} K_{a1,2,3}\\ ⇋ \end{array}\;\;\\ \begin{array}{c} \;\;\\ Ca^{2+}+\mathrm{CA}C^{3-}+3H^{+} \end{array}\;\begin{array}{c} K_{f\;\text{CaCAC}}\\ ⇋ \end{array}\;\begin{array}{c} \;\;\\ \text{CaCA}C^{-}+3H^{+} \end{array} \end{array}$$ (3)

$$\begin{array}{ccc} \begin{array}{c} \;\;\\ \text{HLAC}+Ca^{2+} \end{array} & \begin{array}{c} K_{a}\\ ⇋ \end{array} & \begin{array}{ccc} \begin{array}{c} \;\;\\ \begin{array}{l} Ca^{2+}+\mathrm{LA}C^{-}\\ +H^{+} \end{array} \end{array} & \begin{array}{c} K_{f\;\text{CaLAC}}\\ ⇋ \end{array} & \begin{array}{c} \;\;\\ \begin{array}{l} \text{CaLA}C^{+}\\ +H^{+} \end{array} \end{array} \end{array} \end{array}$$ (4)

$$\begin{array}{c} \;\;\\ Ca_{\text{total}\;\mathrm{WP}}=[Ca^{2+}]+[\text{CaCA}C^{-}]+[\text{CaLA}C^{+}] \end{array}$$ (5)

$$\begin{array}{c} \;\;\\ \begin{array}{l} \mathrm{CA}C_{\text{total}\;\mathrm{WP}}=[H_{3}\mathrm{CAC}]+[H_{2}\mathrm{CA}C^{-}]\\ +[\mathrm{HCA}C^{2-}]+[\mathrm{CA}C^{3-}]+[\text{CaCAC}] \end{array} \end{array}$$ (6)

$$\begin{array}{c} \;\;\\ \mathrm{LA}C_{\text{total}\;\mathrm{WP}}=[\text{HLAC}]+[\mathrm{LA}C^{-}]+[\text{CaLAC}] \end{array}$$ (7)

Where K_a 1,2,3_ of 5.2 · 10^−15^ is the product of the three dissociation constants of citric acid at 25 °C (Budavari, 1989), K_f CaCAC_ of 2,208 is the 1:1 calcium-citrate complex equilibrium constant at 25 °C (Liu et al., 2021), K_a_ of 1.4 · 10^−4^ is the dissociation constant of lactic acid at 25 °C (Budavari, 1989), K_f CaLAC_ of 8.03 is the 1:1 calcium-lactate complex equilibrium constant at 25 °C (Liu et al., 2021), HLAC is the concentration of undissociated lactic acid (M), LAC^−^ the lactate concentration (M), CaLAC^+^ the 1:1 calcium lactate complex concentration (M), Ca^2+^ the calcium concentration (M), H^+^ the hydrogen ion concentration (M), H_3_CAC the concentration of undissociated citric acid (M), CAC^3−^ the citrate concentration (M) and CaCAC^−^ the 1:1 calcium citrate complex concentration (M).

Bound or complexed Ca and free CAC or LAC concentrations were obtained by numerically solving the simultaneous equilibrium and mass-balance equations for each dairy product (Supplementary Table S3) using the nonlinear least-squares solver in SciPy (Virtanen et al., 2020). Exclusively 1:1 stoichiometric complexes were included (Equations 3 and 4), assuming these are most prevalent. Moreover, temperature dependencies and other complexants (e.g., phosphate ions) were not included, although possibly relevant (Jiang et al., 2023; Liu et al., 2021; Hacht, 2007; Walstra et al., 2005).

##### 2.4.2 Evaluation of modified TTT models

The Koukou et al. (2022b) TTT model was modified to exclude the term for interaction between the factors in this model. This modified model was then evaluated for (i) the entire dairy TTT challenge test dataset, that is, including extremities of these data and (ii) the optimized RoA established as described in 2.5.

Furthermore, the Koukou et al. (2022b) TTT model was modified to use total rather than undissociated concentrations of CAC and LAC. This approach was inspired by Maktabdar et al. (2025a, 2025b) where models for psychrotolerant and mesophilic sub-groups of *Bacillus cereus* included terms for total rather than undissociated CAC to obtain unbiased and to avoid fail-dangerous predictions for dairy foods. Equation 8 shows the applied model terms for total CAC and total LAC. *MIC_T CAC_* of 8,150 ± 350 mg CAC/L and *MIC_T LAC_* of 33,442 ± 328 mg LAC/L were fitted from data previously generated by Koukou et al. (2022b) to determine MIC-values for the undissociated acids. This modified model with *MIC_T CAC_* and *MIC_T LAC_* was then evaluated for dairy products within (i) the TTT challenge test dataset extremities and (ii) the optimized RoA established as described in 2.5.

$$\sqrt{\mu_{\max}}=\sqrt{\mu_{\mathrm{ref}-\mathrm{OA}}\cdot {\left(1-{\left(\frac{\mathrm{OA}C_{T}\;}{\mathrm{MI}C_{T\;\mathrm{OA}}\;}\right)}^{n1}\right)}^{n2}}$$ (8)

Where *OAC_T_* is the total concentrations (mM) of, respectively, CAC or LAC. *MIC_T OA_* is the fitted MIC-values (mM) of total organic acids that prevent growth and *μ_ref-OA_* is a fitted parameter that corresponds to *μ_max_* at 25 °C and pH 6.0. To obtain best fit of data, n1 was 1.0 for both acids whereas n2 was 2.0 for CAC and 1.0 for LAC.

##### 2.4.3 Potential evaluation of other TTT models

It was considered to evaluate the TTT models of Tanaka et al. (1986), Ter Steeg and Cuppers (1995) and Glass et al. (2017). The TTT model of Glass et al. (2017) is interesting as it includes effects of both sorbic acid and lipid content in processed cheese. Unfortunately, parameter values of this model are not available and it has therefore not been possible to evaluate its performance as part of the present study. For the models of Tanaka et al. (1986) and Ter Steeg and Cuppers (1995) we were unable to reproduce reported contour plots and therefore did not evaluate these models. Nevertheless, TTT data and product characteristics from Tanaka et al. (1986), Ter Steeg and Cuppers (1995) and Glass et al. (2017) were extracted and included in the present study (Table 3).

**Table 3:** Observed and predicted time-to-toxicity (TTT) for proteolytic *C. botulinum* in dairy food and overview of challenge tests data including product characteristics as extracted from literature.

Product/category	NTO/n ^a^	N_0_ ^b^ (log CFU/g)	Temp. (°C)	WPS ^c^ (%)	a_w_ ^d^	pH	Calcium in WP ^c^ (ppm)	Organic acids in WP ^c^ (mg/L) ^g^		SAC ^g^ in product (mg/kg)	Phosphate ions in WP ^c^ (%) ^g^			Time-to-toxicity (days)		Reference
								CAC	LAC		P1	P2	P3	Observed^h^	Predicted	
Processed cheese (*n* = 256)																
Full fat Cheddar-, one-third reduced fat cheese-, and skim milk fat processed cheeses	0/15	3.0	30	2.0–4.0	0.977–0.989	5.63–5.83	4,009–6,188 ^e^	0	5,594–14,939	0	1.60–1.71	0.00	0.00	4–18	2–25 or NTP ^i^	Glass and Johnson (2004a)
Processed Cheddar cheese	32/83	3.0	27	1.4–7.4	0.954–0.993	5.40–6.20	4,477–5,474 ^e^	0	319–23,330	0-2,100	0.88–2.16	0.00	0.00	4–336	1–26 or NTP ^i^	Glass et al. (2017)
Processed American cheeses with and without garlic, bacon, or citrate	17/29	2.9–3.3	30–35	3.1–4.8	0.972–0.982	5.44–5.80	5,056–5,507 ^e^	0–44,212	976–7,039	0	0.00–5.53	0.00–3.56	0.00	11–217	2–93 or NTP ^i^	Tanaka (1982)
Processed cheese with pimentos	6/9	2.8–3.2	30	3.1–4.2	0.976–0.982	5.80–6.28	3,460–5,150 ^e^	0–44,256	3,381–4,027	0	0.00–3.25	0.00	0.00	14–42	2–4 or NTP ^i^	Tanaka et al. (1979)
Processed palet and “nonfat” cheese mixes	42/108	2.3	15–30	1.7–5.5	0.967–0.991	5.46–6.02	4,669–8,106 ^e^	0–20,697	8,803–26,101	0	0.00–1.64 ^e^	0.00–2.36 ^e^	0.00–0.75 ^e^	2–70	5–55 or NTP ^i^	Ter Steeg et al. (1995)
Processed cheese with 50% fat on dry basis	4/12	3.0	30	4.9–7.3	0.955–0.971	5.66–5.75	7,388 ^e^	0	10,000–26,000	0	0.14 ^e^	0.18 ^e^	0.05 ^e^	2–46	9–265 or NTP ^i^	Ter Steeg and Cuppers (1995)
Cheeses (*n* = 56)																
Surface ripened cheese	4/22	4.0	30	5.0–7.6	0.953–0.971	4.90–6.90	4,136 ^e^	0	1,000 ^e^^,^ ^f^	0	0.00	0.00	0.00	2–45	3–53 or NTP ^i^	Wagenaar and Dack (1958a)
Mold surface ripened cheese	1/19	2.0–4.0	30	5.0–6.5	0.961–0.971	5.10–5.80	4,136 ^e^	0	1,000 ^e^^,^ ^f^	0	0.00	0.00	0.00	2–45	4–658 or NTP ^i^	Wagenaar and Dack (1958b)
Surface ripened cheese	4/15	2.0–4.0	30	3.9–6.4	0.962–0.978	5.30–6.70	4,136 ^e^	0	1,000 ^e^^,^ ^f^	0	0.00	0.00	0.00	5–22	2–13	Wagenaar and Dack (1958c)

a Number of no toxicity observed responses (NTO) and total number of TTT responses (*n*).

b Initial spore concentration (*N*_0_).

c Water phase (WP) and water phase NaCl (WPS).

d The water activity (a_w_) calculated from WPS using: a_w_ = 1–0.0052471 * WPS − 0.00012206 * WPS^2^ (Ross and Dalgaard, 2004).

e Estimated from product characteristics or similar products, as described by Koukou et al. (2022a) and Mejlholm et al. (2010).

f Ripening can notably lower lactic acid concentrations (Martinez-Rios et al., 2020).

g Citric acid (CAC), lactic acid (LAC), sorbic acid (SAC), ortho-phosphate (P1), pyro-phosphate (P2), and tri-phosphate (P3).

h Average of last-time-negative and first-time-positive toxicity observations transformed to days where toxicity was observed.

i No toxicity predicted (NTP).

### 2.5 Determination of optimized ranges of applicability for different models

For both non-dairy and dairy products the studied versions of the Koukou et al. (2022b) TTT model were evaluated for different RoAs (Sections 2.3 and 2.4). This was performed to identify a wide range of environmental factors within which the percentages of fail-dangerous TTT predictions were at most 1.0%. Firstly, each model was evaluated for the full range of product characteristics and storage temperatures (dataset extremities) corresponding to a type of food. Secondly, for fail-dangerous predictions, model terms of environmental factors with values of 0.00 to 0.12 were identified. This pragmatic value of 0.12 was chosen because the quantitative effect in combination with other terms of environmental factors and of interaction between factors was found to result in no-growth predictions. The RoA was then reduced for each of the identified environmental factors to result in <1.0% fail-dangerous TTT predictions and with *ψ*-value of individual fail-dangerous predictions close to one.

### 2.6 Data analysis

Prediction of TTT by the studied models (Sections 2.3 and 2.4) was performed using Microsoft Excel 2016 (Microsoft Corp., Redmond, WA, United States). Binding of Ca with CAC and LAC was simulated using Python (v3.10; Python Software Foundation, 2021) and SciPy (Virtanen et al., 2020). Graphs were prepared with Matplotlib 3.10.0 (Hunter, 2007) in Python v3.10. MIC value for total citric and total lactic acids and their standard errors were fitted using GraphPad PRISM v. 11.0.2 (GraphPad Software, San Diego, CA, USA).

## 3 Results and discussion

### 3.1 Collection of TTT data and product characteristics

To evaluate performance of TTT models a comprehensive dataset has been extracted from 46 challenge tests studies where products were inoculated with spores of individual or cocktails of proteolytic *C. botulinum* strains. These studies reported TTT for a total of 138 different and named proteolytic *C. botulinum* isolates. The generated dataset consisted of observations from 962 treatments including 648 where toxicity was observed and 314 where no-toxicity was detected (Tables 2, 3; Supplementary Tables S1, S2). Storage trials with non-inoculated, that is, naturally contaminated, products were not found and therefore not included in the present study. This is unfortunate, as those data, with very low initial spore concentrations, would have been most relevant to evaluate the performance of predictive models as previously observed for other foodborne pathogens (Mejlholm et al., 2010, 2015; Maktabdar et al., 2025b). Storage time for the studied challenge tests was from one to 392 days and observed TTT varied from 0.5 to 336 days. Product characteristics included the following: temperature (4–35 °C), packaging in air, vacuum or modified atmospheres with <0.3% O_2_, <80% CO_2_ and 20–100% N_2_, dry matter (0.3–85%), lipid (0–58%), pH (4.4–8.5), WPS (0.0–9.5%), measured a_w_ (0.920–0.994), water phase concentrations of AAC (≤64,364 mg/L), BAC (≤918 mg/L), CAC (≤65,459 mg/L), LAC (≤70,340 mg/L) and SAC (≤2,119 mg/L), water phase concentrations of P1 (≤5.53%), P2 (≤3.56%) and P3 (≤0.75%), added sodium nitrate (≤340 mg/kg in product) and Ca (≤4,934 mg/kg in product) (Tables 2, 3; Supplementary Tables S1, S2). Data were collected from challenge tests where no dynamic changes in storage temperature or product characteristics were reported during the storage time. Except for seafood, no TTT data was collected from challenge tests with products reported to contain BAC. P3 concentrations for the collected data were consistently below 0.83% in water phase and therefore had no inhibitory effect on TTT as predicted by the Koukou et al. (2022b) TTT model (Section 2.2). The lowest storage temperature with observed TTT was 12 °C (Larson et al., 1997).

The compiled dataset with TTT responses and product characteristics is comprehensive and allowed evaluation and comparison of performance for TTT models with different foods representing a broad range of envitonmental conditions. Datasets for TTT responses of proteolytic *C. botulinum* were previously generated, for example, for pork slurry (Roberts et al., 1981, 1982), processed cheese (Tanaka, 1982; Tanaka et al., 1986; Ter Steeg et al., 1995; Glass et al., 2017), and potatoes and crabmeat [International Commission on Microbiological Specifications for Foods (ICMSF), 1996]. Importantly, the dataset compiled in the present study includes data from several of those previous studies. In addition, a substantial effort was made to collect both TTT responses and numerous product characteristics to allow evaluation of model performance in the present study.

### 3.2 Expanding the growth model to predict TTT

The average increase in cell concentrations of proteolytic *C. botulinum* that corresponded to TTT formation (*ΔN_TTT_*) was 129 CFU/g and log(*ΔN_TTT_*) was 2.11 ± 0.79 (Table 1). For proteolytic *C. botulinum*, Ter Steeg and Cuppers (1995), previously, used a value of 2.0 for log(*ΔN_TTT_*) as a conservative TTT estimator and for non-proteolytic *C. botulinum* a log(*ΔN_TTT_*)-value of 2.2 ± 1.3 has been determined (Koukou et al., 2021). A new extensive model to predict TTT for proteolytic *C. botulinum* was obtained by using the estimated *ΔN_TTT_* -value in Equation 3 together with lag times and *μ_max_*-values as predicted by the available growth and growth boundary model of Koukou et al. (2022b) depending on product characteristics and storage conditions. The new Koukou et al. (2022b) TTT model has the potential to predict TTT for a wide range of environmental factors. For model evaluation, a limitation of this model is that TTT cannot be predicted for initial spore concentrations higher than 4.7 log CFU/g corresponding to the difference between the model’s maximum population density of 6.8 ± 1.0 log CFU/g, (Section 2.2) and log(*ΔN_TTT_*). However, *μ_max_*-values are predicted for initial spore concentrations higher than 4.7 log CFU/g and if *μ_max_* was >0 it was assumed that toxin eventually will be formed. These qualitatively predicted TTT responses were therefore included when calculating the percentage of correct, fail-safe and fail-dangerous predictions but not to calculate values of B_f-TTT_ and A_f-TTT_ (Tables 4–7).The large majority of studies where proteolytic *C botulinum* formed toxin in foods did not report corresponding growth kinetics. We found just 15 kinetics where both TTT and growth were reported (Table 1). These kinetics support that toxin formation follow spore germination and outgrowth of metabolically active vegetative cells (Bonventre and Kempe, 1960; Connan et al., 2013; Rawson et al., 2023). We found no studies where proteolytic *C. botulinum* did not grow but formed toxin in food. Lövenklev et al. (2004) observed that introduction of air to a vegetative culture of proteolytic *C. botulinum* strain ATTC 7949, at the end of the exponential growth phase, can result in type B toxin formation, despite a decrease in viable counts. However, this was observed at a cell density (OD_620_ > 2.0) much higher than found for spores in naturally contaminated foods prior to germination.

**Table 4:** Performance of Koukou et al. (2022b) TTT model for proteolytic *C. botulinum* in non-dairy foods.

Environmental factors and performance indices	Koukou et al. (2022b) TTT model		
	(i) TTT challenge test dataset extremities	(ii) Range of applicability by Koukou et al. (2022b)	(iii) Optimized non-dairy range of applicability
Temperature (°C)	4.0–35.0	11.9–37	4.0–35.0
pH	4.40–8.50	5.65–7.20	5.10–8.50
Water phase NaCl (%)	≤9.5	<10.8	≤9.5
Water phase acetic acid (mg/L)	≤65,364	<8,277	<8,277
Water phase benzoic acid (mg/L)	≤918	<190	≤881
Water phase citric acid (mg/L)	≤65,459	<3,752	≤1,226
Water phase lactic acid (mg/L)	≤70,340	<16,980	≤23,539
Water phase sorbic acid (mg/L)	≤2,119	<261	≤1,117
Water phase ortho-phosphate (%)	≤0.00	<4.91	≤0.00
Water phase pyro-phosphate (%)	≤0.43	<1.05	≤0.43
Water phase tri-phosphate (%)	≤0.59	<1.07	≤0.59
Non-dairy foods (meat-, poultry-, vegetable-, dough-, seafood products, and uncategorized foods)			
*n*	650	359	494
Fail-dangerous, *n* (%)	38 (5.8%)	1 (0.3%)	1 (0.2%)
Fail-safe, *n* (%)	98 (15.1%)	67 (18.7%)	91 (18.4%)
*n*_f_ ^a^	301	209	281
Bias factor-TTT	0.53	0.48	0.50
Accuracy factor-TTT	2.55	2.53	2.54
Meat products with and without nitrite			
*n*	233	143	179
Fail-dangerous, *n* (%)	15 (6.4%)	1 (0.7%)	1 (0.6%)
Fail-safe, *n* (%)	22 (9.4%)	15 (10.5%)	17 (9.5%)
*n*_f_ ^a^	81	71	81
Bias factor-TTT	0.50	0.43	0.50
Accuracy factor-TTT	2.62	2.73	2.62
Poultry products with and without nitrite			
*n*	77	49	52
Fail-dangerous, *n* (%)	5 (6.5%)	0 (0.0%)	0 (0.0%)
Fail-safe, *n* (%)	11 (14.3%)	11 (22.4%)	11 (21.2%)
*n*_f_ ^a^	57	38	41
Bias factor-TTT	0.58	0.53	0.56
Accuracy factor-TTT	1.96	2.05	2.02
Seafoods			
*n*	107	41	45
Fail-dangerous, *n* (%)	16 (15.0%)	0 (0.0%)	0 (0.0%)
Fail-safe, *n* (%)	8 (7.5%)	4 (9.8%)	6 (13.3%)
*n*_f_ ^a^	35	31	31
Bias factor-TTT	1.55	1.24	1.24
Accuracy factor-TTT	2.19	1.75	1.75
Dough products			
*n*	34	20	32
Fail-dangerous, *n* (%)	0 (0.0%)	0 (0.0%)	0 (0.0%)
Fail-safe, *n* (%)	11 (32.4%)	6 (30.0%)	11 (34.4%)
*n*_f_ ^a^	15	8	15
Bias factor-TTT	0.46	0.28	0.46
Accuracy factor-TTT	2.99	3.87	2.99
Vegetables			
*N*	149	89	138
Fail-dangerous, *n* (%)	2 (1.3%)	0 (0.0%)	0 (0.0%)
Fail-safe, *n* (%)	32 (21.5%)	30 (32.6%)	32 (23.2%)
*n*_f_ ^a^	84	49	84
Bias factor-TTT	0.50	0.39	0.50
Accuracy factor-TTT	2.33	2.70	2.33
Uncategorized foods			
*N*	50	14	48
Fail-dangerous, *n* (%)	0 (n = 0.0%)	0 (n = 0.0%)	0 (n = 0.0%)
Fail-safe, *n* (%)	14 (28.0%)	6 (42.9%)	14 (29.2%)
*n*_f_ ^a^	29	12	29
Bias factor-TTT	0.19	0.24	0.19
Accuracy factor-TTT	5.66	4.77	5.66

Time-to-toxicity (TTT) were predicted using undissociated concentrations of organic acids and model input and for three different ranges of product characteristics.

a Number of treatments where time-to-toxicity (TTT) were numerically both observed and predicted to allow calculation of bias- and accuracy factor values.

**Table 5:** Performance of Koukou et al. (2022b) TTT model for proteolytic *C. botulinum* in dairy foods.

Environmental factors and performance indices	Koukou et al. (2022b) TTT model		Koukou et al. (2022b) TTT model with calcium-citrate/lactate complexation correction	
	(i) TTT challenge test dataset extremities	(ii) Range of applicability by Koukou et al. (2022b)	(iii) TTT challenge test dataset extremities	(iv) Optimized dairy range of applicability
Temperature (°C)	15.0–35.0	11.9–37.0	15.0–35.0	11.9–35.0
pH	4.90–6.90	5.65–7.20	4.90–6.90	5.20–6.90
Water phase NaCl (%)	≤7.6	<10.8	≤7.6	<10.8
Water phase acetic acid (mg/L)	≤0	<8,277	≤0	≤8,277
Water phase benzoic acid (mg/L)	≤0	<190	≤0	≤190
Water phase citric acid (mg/L)	≤44,256	<3,752	≤44,256	≤5,660
Water phase lactic acid (mg/L)	≤26,101	<16,980	≤26,101	≤11,443
Water phase sorbic acid (mg/L)	≤1,658	<261	≤1,658	≤1,633
Water phase orthophosphate (%)	≤5.53	<4.91	≤5.53	≤5.53
Water phase pyrophosphate (%)	≤3.56	<1.05	≤3.56	≤3.56
Water phase triphosphate (%)	≤0.75	<1.07	≤0.75	≤1.07
Dairy foods (processed cheeses and cheeses)				
*n*	312	80	312	144
Fail-dangerous, *n* (%)	81 (26.0%)	3 (3.8%)	34 (10.9%)	1 (0.7%)
Fail-safe, *n* (%)	33 (10.6%)	15 (18.8%)	38 (12.2%)	35 (24.3%)
*n*_f_ ^a^	121	62	168	101
Bias factor-TTT	0.55	0.44	0.74	0.37
Accuracy factor-TTT	4.37	3.60	4.66	4.58
Processed cheeses				
*n*	256	61	256	97
Fail-dangerous, *n* (%)	76 (29.7%)	3 (4.9%)	30 (11.7%)	1 (1.0%)
Fail-safe, *n* (%)	25 (9.8%)	8 (13.1%)	30 (11.7%)	28 (28.9%)
*n*_f_ ^a^	79	50	125	61
Bias factor-TTT	0.33	0.45	0.57	0.17
Accuracy factor-TTT	5.10	3.76	5.14	6.34
Cheeses				
*n*	56	19	56	47
Fail-dangerous, *n* (%)	5 (8.9%)	0 (0.0%)	4 (7.1%)	0 (0.0%)
Fail-safe, *n* (%)	8 (14.3%)	7 (36.8%)	8 (14.3%)	7 (14.9%)
*n*_f_ ^a^	42	12	43	40
Bias factor-TTT	1.45	0.41	1.54	1.16
Accuracy factor-TTT	3.28	3.01	3.51	2.79

Time-to-toxicity (TTT) was predicted without or with correction for binding of citrate and lactate by calcium.

a Number of treatments where time-to-toxicity (TTT) were numerically both observed and predicted to allow calculation of bias- and accuracy factor values.

**Table 6:** Performance of modified Koukou et al. (2022b) TTT models for proteolytic *C. botulinum* in dairy foods.

Environmental factors and performance indices	Koukou et al. (2022b) TTT model without interaction		Koukou et al. (2022b) TTT model with total concentrations of citric and lactic acids	
	(i) TTT challenge test dataset extremities	(ii) Optimized dairy range of applicability	(iii) TTT challenge test dataset extremities	(iv) Optimized dairy range of applicability
Temperature (°C)	15.0–35.0	11.9–35.0	15.0–35.0	11.9–35.0
pH	4.90–6.90	5.20–6.90	4.90–6.90	5.20–6.90
Water phase NaCl (%)	≤7.6	<10.8	≤7.6	<10.8
Water phase acetic acid (mg/L)	≤0	≤8,277	≤0	≤8,277
Water phase benzoic acid (mg/L)	≤0	≤190	≤0	≤190
Water phase citric acid (mg/L)	≤44,256	≤5,660	≤44,256	≤5,660
Water phase lactic acid (mg/L)	≤26,101	≤11,443	≤26,101	≤11,443
Water phase sorbic acid (mg/L)	≤1,658	≤1,633	≤1,658	≤1,633
Water phase orthophosphate (%)	≤5.53	≤5.53	≤5.53	≤5.53
Water phase pyrophosphate (%)	≤3.56	≤3.56	≤3.56	≤3.56
Water phase triphosphate (%)	≤0.75	≤1.07	≤0.75	≤1.07
Dairy foods (processed cheeses and cheeses)				
*n*	312	144	312	144
Fail-dangerous, *n* (%)	56 (17.9%)	2 (1.4%)	44 (14.1%)	0 (0.0%)
Fail-safe, *n* (%)	38 (12.2%)	36 (25%)	46 (14.7%)	34 (23.6%)
*n*_f_ ^a^	146	100	158	102
Bias factor-TTT	0.92	0.44	0.49	0.37
Accuracy factor-TTT	5.32	4.51	4.09	4.42
Processed cheeses				
*n*	256	97	256	97
Fail-dangerous, *n* (%)	55 (21.5%)	2 (2.1%)	40 (15.6%)	0 (0.0%)
Fail-safe, *n* (%)	30 (11.7%)	29 (29.9%)	38 (14.8%)	27 (27.8%)
*n*_f_ ^a^	100	60	115	62
Bias factor-TTT	0.75	0.23	0.33	0.18
Accuracy factor-TTT	6.88	6.29	4.54	6.05
Cheeses				
*n*	56	47	56	47
Fail-dangerous, *n* (%)	1 (1.8%)	0 (0.0%)	4 (7.1%)	0 (0.0%)
Fail-safe, *n* (%)	8 (14.3%)	7 (14.9%)	8 (14.3%)	7 (14.9%)
*n*_f_ ^a^	46	40	43	40
Bias factor-TTT	1.45	1.17	1.36	1.12
Accuracy factor-TTT	3.04	2.74	3.10	2.72

Time-to-toxicity (TTT) was predicted without the effect of interaction between factors in model or by using total rather than undissociated concentration of citric and lactic acids.

a Number of treatments where time-to-toxicity (TTT) were numerically both observed and predicted to allow calculation of bias- and accuracy factor values.

### 3.3 Evaluation of the Koukou model expanded to predict TTT

#### 3.3.1 *Effect of* food matrices and initial spore concentrations on performance of TTT model

When evaluated using the entire TTT dataset collected, the new Koukou et al. (2022b) TTT model performed markedly better for non-dairy than for dairy products (Table 7). Dairy products had both higher percentages of fail-dangerous TTT predictions (26.0% vs. 5.8%) and higher A_f-TTT_–values (4.37 vs. 2.55) than non-dairy foods. This suggests that the two product categories should be evaluated separately. For non-dairy foods, values of the studied indices for model performance did not seem to correlate with initial spore concentrations (Tables 2, 7). Some other studies found the probability of toxin formation by proteolytic *C. botulinum* to increase at higher initial spore concentrations, for example, for pork slurry (Roberts et al., 1981; Robinson et al., 1982). As one example for unheated pork slurry with 3.5% WPS, 200 mg/kg sodium nitrite and pH 5.54–6.36, the probability of toxin formation after 180 days at 15 °C was 3% with 0.36 spores/ml and 15% with 36 spores/ml (Roberts et al., 1981). For the studied dairy foods, the percentage of fail-dangerous predictions of toxin formation seemed to decrease at higher initial spore concentrations (Table 7). This corresponds to the effect of initial spore concentrations observed by Roberts et al. (1981). However, few dairy challenge tests had initial spore concentrations <2 and >4 log spores/g (Table 7). Furthermore, Ter Steeg et al. (1995) found TTT of 8 weeks in processed cheese at 25 °C with initial spore concentrations from 0.1/g to 10/g and just a little shorter TTT (7 weeks) for initial spore concentrations of 90/g and 770/g. More studies with low initial spore concentrations seem relevant as this will resemble naturally contaminated foods whereas high initial spore concentrations are of little relevance for safety of real foods (Dodds, 1994; Anderson et al., 2011; Lindström et al., 2010; Pernu et al., 2020).

**Table 7:** Indices of predicted^a^ and observed toxin formation for non-dairy and dairy foods as influenced by initial spore concentrations of proteolytic *C. botulinum* and by method for water activity determination^b^.

Water activity ^b^	N_0_ ^c^ (log CFU/g)	Indices for qualitative toxin formation				Indices for quantitative TTT		
		Total ^d^	Correct	Fail-safe	Fail-dangerous	n_f_ ^e^	B_f-TTT_^e^	A_f-TTT_^e^
Non-dairy foods (meat-, poultry-, vegetable-, dough-, seafood, and uncategorized foods)								
a_w_-values estimated from % WPS used as model input^f^	−1 ≤ N₀ < 1	61 (100%)	51 (84%)	7 (11%)	3 (5%)	12	0.68	1.72
	1 ≤ N₀ < 3	348 (100%)	265 (76%)	54 (16%)	29 (8%)	164	0.51	2.81
	3 ≤ N₀ < 5	207 (100%)	171 (83%)	32 (15%)	4 (2%)	125	0.55	2.32
	5 ≤ N₀ < 7	34 (100%)	27 (79%)	5 (15%)	2 (6%)	0	-	-
	−1 ≤ N₀ < 7	650 (100%)	514 (79.1%)	98 (15.1%)	38 (5.8%)	301	0.53	2.55
Measured a_w_-values used as model input	−1 ≤ N₀ < 1	61 (100%)	51 (84%)	7 (11%)	3 (5%)	12	0.96	1.64
	1 ≤ N₀ < 3	348 (100%)	269 (77%)	48 (14%)	31 (9%)	162	0.63	2.30
	3 ≤ N₀ < 5	207 (100%)	174 (84%)	29 (14%)	4 (2%)	125	0.74	2.39
	5 ≤ N₀ < 7	34 (100%)	27 (79%)	5 (15%)	2 (6%)	0	-	-
	-1 ≤ N₀ < 7	650 (100%)	521 (80.2%)	**89 (13.7%)**	40 (6.2%)	299	0.69	2.31
Dairy foods (processed cheeses and other cheeses)								
a_w_-values estimated from % WPS used as model input ^f^	0 ≤ N₀ < 2	0	-	-	-	0	-	-
	2 ≤ N₀ < 4	265 (100%)	163 (62%)	25 (9%)	77 (29%)	87	0.36	4.86
	4 ≤ N₀ < 5	47 (100%)	35 (74%)	8 (17%)	4 (9%)	34	1.65	3.33
	0 ≤ N₀ < 5	312 (100.0%)	198 (63.5%)	33 (10.6%)	81 (26.0%)	121	0.55	4.37
Measured a_w_-values used as model input	0 ≤ N₀ < 2	0	-	-	-	0	-	-
	2 ≤ N₀ < 4	265 (100%)	169 (64%)	9 (3%)	87 (33%)	77	0.85	3.44
	4 ≤ N₀ < 5	47 (100%)	35 (74%)	8 (17%)	4 (9%)	34	1.65	3.33
	0 ≤ N₀ < 5	312 (100.0%)	204 (65.4%)	17 (5.4%)	91 (29.2%)	111	1.04	3.41

a Qualitative toxin formation and quantitative time-to-toxicity (TTT) were predicted by the Koukou et al. (2022b) TTT model.

b Water activity input to the Koukou et al. (2022b) TTT model were evaluated for both (i) a_w_-values estimated from the percentage of water phase NaCl in products and as (ii) measured a_w_-values in products.

c Initial spore concentration.

d Total number of instances where toxin formation was qualitatively observed and predicted.

e Number of numeric time-to-toxicity observations and predictions used for calculation of bias-factor (B_f-TTT_) and accuracy-factor (A_f-TTT_) values.

f The Koukou et al. (2022b) growth and growth boundary model was developed using a_w_-values, calculated from NaCl in the water phase, as model input (Section 2.2).

The Koukou et al. (2022b) growth and growth boundary model was developed using a_w_-values, calculated from WPS, as model input (Section 2.2). For the entire TTT dataset it had little effect on the percentages of fail-dangerous TTT predictions in non-dairy (5.8% vs. 6.2%) or dairy (26.0% vs. 29.2%) foods if a_w_-values were estimated from WPS or measured directly in products (Table 7). However, particularly for dairy foods B_f-TTT_ -values were lower and the A_f-TTT_-values higher when calculated from predictions based on a_w_-values estimated from WPS compared to a_w_-values measured directly in products (Table 7). These very high A_f-TTT_-values of 4.37 and 3.41 showed pronounced differences between predicted and observed TTT but A_f-TTT_-values for the two types of a_w_-values cannot be directly compared as the corresponding B_f-TTT_-values differed. Therefore, the *μ_ref_*-value of models used for the four scenarios shown in Table 7 were all calibrated to result in B_f-TTT_-values of 1.00. Accordingly, A_f-TTT_ values of 2.14 and 2.18 were obtained for non-dairy foods using a_w_ values estimated from WPS and measured directly in products, respectively. The corresponding A_f-TTT_-values for dairy foods were 4.07 and 3.42 (Results not shown).

These results suggest pronounced difference between predicted and observed TTT values for both non-dairy and dairy foods. Consequently, in the present study, we analyzed model performance for sub-groups of both of these types of foods. Furthermore, the model’s RoA was reduced to evaluate how this influenced model performance. A_w_-values estimated from WPS were used as input to the new Koukou et al. (2022b) TTT model and the *μ_ref_*-value of this model was not calibrated for types or sub-groups of the products studied (Tables 4–6).

#### 3.3.2 Evaluation of model performance for non-dairy foods

TTT data collected for non-dairy products resulted from 131 different isolates of proteolytic *C. botulinum* as used in challenge tests with a wide range of product characteristics, except for the three phosphate ions. Storage of products in air, VP and MAP were represented. For the full range of these data (dataset extremities; *n* = 650), the Koukou et al. (2022b) TTT model provided 38 (5.8%) fail-dangerous TTT predictions and several of the predictions were not close to the growth boundary (*ψ* -values of 1.15 to 78.8). This suggests the Koukou et al. (2022b) TTT model was not accurate for the full data range and/or that product characteristics for some of the studied foods were not precise (Tables 2, 4; Supplementary Table S1).

Meat and poultry products included treatments with added sodium nitrite. Meat products without or with added sodium nitrite had B_f-TTT_-/A_f-TTT_-values of, respectively, 0.95/1.91 calculated from 36 challenge tests (*n*_f_) and 0.30/3.37 (*n*_f_ = 45). For poultry products the corresponding values were 0.87/1.71 (*n*_f_ = 16) and 0.48/2.09 (*n*_f_ = 39; Results not shown). Thus, sodium nitrite added to meat and poultry products on average extended TTT by factors of, respectively, 3.1 and 1.8. Some of the studied sodium nitrite concentrations (≤340 mg/kg in product) were higher than the EU limit of 120 mg/kg [EU (European Commission), 2023]. However, these results illustrate the inhibiting effect of nitrite that other factors must be designed to manage in nitrite free recipes.

Lipid content in products markedly reduced both the observed and the predicted inhibiting effect of SAC. Of the 301 non-dairy products where TTT were both observed and predicted (B_f-TTT_ = 0.53), 26 products contained SAC (117–2,105 mg/kg in products) and 0.5 to 52.0% lipid (Supplementary Table S1). For these products the Koukou et al. (2022b) TTT model predicted zero (0.0%) fail-dangerous responses and B_f-TTT_ was 0.52 (*n*_f_ = 26). However, when excluding the effect of lipid content in products on calculated water phase SAC concentrations, the corresponding predictions resulted in 15 fail-dangerous responses (63%) and B_f-TTT_ was 0.71 (*n*_f_ = 11; Results not shown). In the same way, Glass and Johnson (2004b) found increased fat content in processed cheese to reduce the anti-botulinum activity of SAC. It may be relevant to note that the present study, like Koukou et al. (2022b) for proteolytic *C. botulinum* and Maktabdar et al. (2025b) for *Bacillus cereus*, used two steps to calculate firstly SAC concentrations in water phase of products (Equation 1) and then undissociated SAC concentrations in the water phase. The one-step calculation suggested by Brocklehurst and Wilson (2000) resulted in higher undissociated SAC concentrations in the water phase of products, more fail-dangerous predictions of toxin formation and it was not applied in the present study (Results not shown).

**Figure 1:**
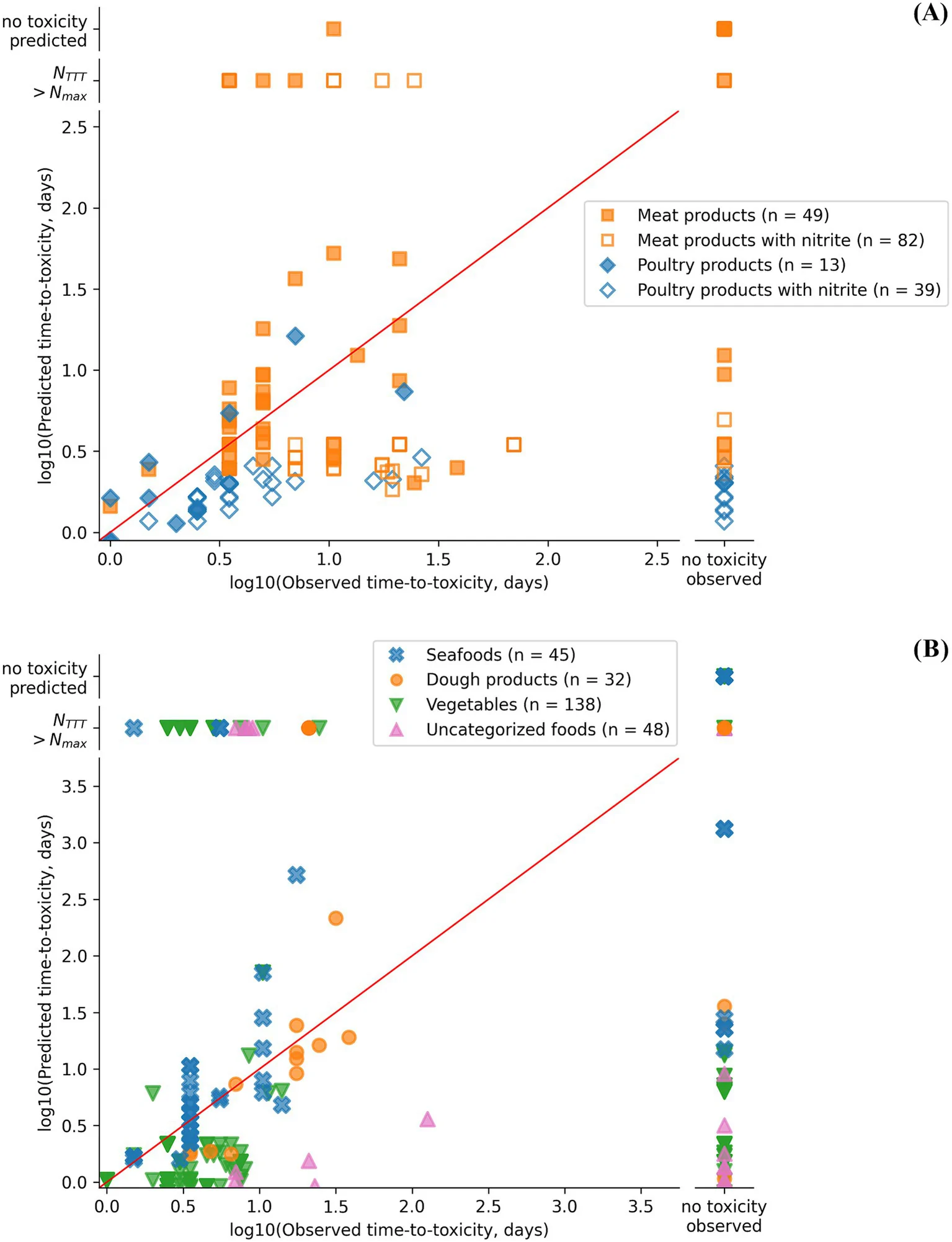
Observed and predicted time-to-toxicity (TTT) for proteolytic *C. botulinum* in challenge tests with meat products, meat products with nitrite, poultry products, poultry products with nitrite **(A)**, seafood, dough products, vegetables, and uncategorized products **(B)**. Predictions were performed within the optimized range of applicability (Table 4). Numeric values on the “no toxicity predicted” axis indicate fail-dangerous predictions, whereas points at the intersection of the no-toxicity observed and predicted axes indicate correct predictions. Conditions in which the predicted population density at the observed time of toxin formation corresponded to N_max_ are shown on the N_TTT_ > N_max_ axis as no quantitatively predicted time for toxin formation was obtained.

When used exclusively within its RoA, as previously determined from growth responses in processed cheese and meat products, the Koukou et al. (2022b) TTT model provided one (0.3%) fail-dangerous TTT prediction and B_f-TTT_-/A_f-TTT_-values were 0.48/2.53 (*n*_f_ = 209; Table 4). Compared to the full range of environmental factors for non-dairy foods (Dataset extremities; Table 4) a new and optimized RoA, with one (0.2%) fail-dangerous TTT prediction (ID 105; *Ψ* = 1.15; Initial spore concentration = 2.6 log spores/g; Supplementary Table S1), was obtained for this model by (i) reducing the pH range from 4.40–8.50 to 5.10–8.50), (ii) reducing water phase AAC from ≤65,364 mg/L to ≤8,277 mg/L, (iii) reducing water phase BAC from ≤918 mg/L to ≤881 mg/L, (iv) reducing water phase CAC from ≤65,459 mg/L to ≤1,226 mg/L, (v) reducing water phase LAC from ≤70,340 mg/L to ≤23,539 mg/L, and (vi) reducing water phase SAC from ≤2,119 mg/L to ≤1,117 mg/L (Table 4). This optimized non-dairy RoA still included a wide range of environmental factors where the Koukou et al. (2022b) TTT model had 81.4% correct and 18.4% fail-safe qualitative TTT predictions. The improved model performance for the optimized non-dairy RoA compared to the full range of environmental factors (Dataset extremities with 79.1% correct, 15.1% fail-safe and 5.8% fail-dangerous qualitative TTT predictions) suggest predictions by the Koukou et al. (2022b) TTT model are less precise at low pH and with high concentrations of AAC, BAC, CAC, LAC and SAC. Within the new and optimized RoA, the TTT model of Koukou et al. (2022b) provided B_f-TTT_- /A_f-TTT_-values of 0.50/2.54 (*n* = 281; Table 4). Koukou et al. (2022b) found their model to predict growth and growth boundary of proteolytic *C. botulinum* in meat products with good performance (0.0% fail-dangerous, n = 84; B_f-μmax_/A_f-μmax_-values of 1.01/1.58, *n* = 78). The present study found 0.2% fail-dangerous TTT predictions for a much larger dataset (*n* = 494) including six sub-groups of non-dairy food products (Table 4). This is important and indicates that the Koukou et al. (2022b) model, within its new and optimized RoA, can be used to predict combinations of product characteristics and storage temperatures that prevent growth and toxin formation by proteolytic *C. botulinum* in various non-dairy food products. This application of the model is further explained in section 3.4. However, B_f-TTT_ values for meat and poultry products with nitrite, seafoods, vegetables and uncategorized products were <0.7 or >1.15 and A_f-TTT_-values for all studied sub-categories of food products were high (1.8–5.7; Table 4). Due to this variability between observed and predicted TTT, also shown in Figure 1 and Table 2, it should not be attempted to predict more exact TTT values using the Koukou et al. (2022b) TTT model.

Very few studies previously evaluated the performance of TTT or growth models for proteolytic *C. botulinum* in non-dairy foods. Models for growth of proteolytic *C. botulinum* at different temperatures have been evaluated for product cooling profiles and they were found to perform acceptably for different specific uncured meat products (Juneja and Marks, 1999; Juneja et al., 2021a, 2021b; Juneja et al., 2022). Torres-Baix et al. (2023) used the Combase Predictor (www.combase.cc) and found substantial predicted growth during about 15 months of processing for dry-cured ham (salting, resting, drying and ripening). Growth of proteolytic *C. botulinum* was not measured to evaluate the predictions. However, measured dynamic product characteristics were evaluated and this suggests predicted growth was much too fast as the product otherwise should be expected, at least occasionally, to cause botulism which has not been observed (Torres-Baix et al., 2023; Advisory Committee on the Microbiological Safety of Food (ACMSF), 2023). Golden et al. (2017) showed the model of Schaffner et al. (1998) to predict much shorter TTT than observed for Dijon pork and cauliflower mashed potatoes but they did not calculate indices of model performance. Models for probability of toxin formation by proteolytic *C. botulinum* have been developed by fitting experimental data, e.g., obtained for canned comminuted pork (Ivey and Robach, 1978), pork slurry (Roberts et al., 1981, 1982; Robinson et al., 1982) or cooked vacuum-packed potatoes (Dodds, 1989). Evaluation of these models with data from other independent studies has not been reported. Compared to the importance of proteolytic *C. botulinum* for food safety, the previous evaluation of models to predict TTT or growth in non-dairy foods seems limited and the present study represents substantial progress (Table 4; Figure 1). Firstly, by determination of a range of product characteristics and storage conditions, that is, the optimized RoA for non-dairy foods, where the Koukou et al. (2022b) model can be used to predict combinations of product characteristics and storage temperatures that prevent growth and toxin formation. Secondly, by documenting TTT responses and variability of these data for non-dairy foods.

The considerable variability between observed and predicted TTT in non-dairy foods (A_f-TTT_ = 2.54; *n*_f_ = 281) reflected an underestimation of TTT by the Koukou et al. (2022b) TTT model (B_f-TTT_ = 0.50) for all studied sub-groups except seafood (Table 4). To evaluate variability between observed and predicted TTT without this bias, the Koukou et al. (2022b) TTT model was used for each sub-group of non-dairy foods with *μ_ref_*-values resulting in B_f-TTT_ = 1.00 for the sub-group. This provide A_f-TTT_ of 1.93 (*n*_f_ = 281) for non-dairy foods. A_f-TTT_/*μ_ref_*-values (1/h) for product sub-groups were: meat (2.34/1.25), poultry (1.50/1.39), seafood (1.78/3.10), dough products (2.39/1.15), vegetables (1.76/1.25) and uncategorized foods (2.00/0.475) (Results not shown). This suggests TTT-predictions with acceptable A_f-TTT_-values, as a measure of variability, can be obtained for sub-groups of non-dairy foods by calibrating exclusively the *μ_ref_*-values in the Koukou et al. (2022b) TTT model. These sub-groups specific TTT-models have *μ_ref_*-values calibrated using all data for each product sub-group and are therefore not validated. In addition to effects of food matrices, numerous other factors are likely to influence the apparent variability of TTT in non-dairy foods. Further research, including among other aspects studies of well characterized products, seems required before validated models to accurately predict TTT can be developed.

#### 3.3.3 Evaluation of model performance for dairy foods

TTT data were collected for dairy foods (*n* = 312) with a wide range of product characteristics and storage at 15–35 °C in vacuum packaging, anaerobic or unspecified conditions. For these products the lipid content varied from 0.1 to 41.2%. Twenty-two different proteolytic *C. botulinum* isolates were used for inoculation of these challenge tests (Tables 3, 5, Extremities of TTT dataset; Supplementary Table S2). Concentrations of AAC and BAC were not reported. It is, however, most unlikely that the studied processed and other cheeses did not contain AAC as up to 2,397 mg/L in water phase have been determined for industrially produced processed cheeses (Martinez-Rios et al., 2019; Koukou et al., 2022b; Maktabdar et al., 2025b).

##### 3.3.3.1 Evaluation of the TTT model

Even without including the potential growth inhibiting effect of AAC in the studied dairy foods, the Koukou et al. (2022b) TTT model provided 26.0% fail-dangerous and 10.6% fail-safe TTT predictions as previously mentioned (Tables 7, 5). Unacceptably high percentages of fail-dangerous TTT predictions were obtained for both processed cheese (29.7%) and other cheeses (8.9%; Table 5). When used exclusively within its RoA, as determined from growth responses, the Koukou et al. (2022b) TTT model provided 3.8% fail-dangerous and 18.8% fail-safe TTT predictions (n = 80) and B_f-TTT_-/A_f-TTT_-values were 0.44/3.60 (*n*_f_ = 62; Table 5). In validation studies with processed cheese, Koukou et al. (2022b) found 0.0% fail-dangerous growth predictions (*n* = 28) and B_f-μmax_/A_f-μmax_-values of 1.17/1.87 (*n*_f_ = 20). The difference between 3.8 and 0.0% fail-dangerous predictions for, respectively, TTT and growth responses may relate to growth being studied for one cocktail of three *C. sporogenes* isolates in well characterized processed cheeses whereas TTT responses were obtained from several studies including 22 different *C. botulinum* isolates and where concentrations of environmental factors may have been less accurately determined (Table 3; Supplementary Table S2). However, this situation was similar for meat products where the percentage of fail-dangerous predictions were more alike for TTT (0.7%, n = 143) and growth (0.0%, *n* = 84; Table 4; Koukou et al., 2022b). Interestingly, 55 of the 81 fail-dangerous TTT predictions observed for the full range of environmental factors in the studied dairy foods (*n* = 312; Table 5) were directly due to undissociated concentrations of CAC or LAC being higher than their MIC-values. This resulted in prediction of no growth and a value of zero for the model term of each of these two acids although toxin formation was recorded (Supplementary Table S2). Clearly, the Koukou et al. (2022b) TTT model markedly over-estimated the growth inhibiting effects of CAC and LAC in dairy foods. For non-dairy foods this was observed to a much lesser extent (Tables 4, 5).

##### 3.3.3.2 Effect of calcium on predicted TTT responses in dairy foods

Ca in dairy products reduced the calculated concentrations of free CAC and of free LAC by respectively, 90.1 ± 10.1% and 39.6 ± 11.5% (Supplementary Table S3). When using these calculated concentrations of unbound CAC and LAC, the percentage of fail-dangerous TTT predictions obtained by the Koukou et al. (2022b) TTT model was reduced from 26.0 to 10.9% whereas the percentage of fail-safe predictions increased from 10.6 to 12.2% (*n* = 312; Table 5). B_f-TTT_- /A_f-TTT_-values were 0.74/4.7 (*n*_f_ = 168; Table 5). This suggests complexation between Ca and each of the two organic acids is an important part of the explanation why toxin formation in dairy foods was poorly predicted by the Koukou et al. (2022b) TTT model when used without this complexation effect (Section 3.3.3). With Ca correction and an optimized RoA, the Koukou et al. (2022b) TTT model provided one (0.7%) fail-dangerous and 35 (24.3%) fail-safe TTT predictions (*n* = 144). B_f-TTT_−/A_f-TTT_-values were 0.37/4.6 (*n*_f_ = 101; Table 5). The single fail-dangerous TTT prediction was very close to the growth boundary (*ψ*-value of 1.12) and due to processed cheese inoculated with 1,000 spores/g (ID 743; Supplementary Table S2). The optimized RoA for the model used with Ca complexation was obtained by (i) reducing the pH range from 4.90–6.90 to 5.20–6.90), (ii) reducing water phase CAC from ≤44,256 mg/L to ≤5,660 mg/L, and (iii) reducing water phase LAC from ≤26,101 mg/L to ≤11,443 mg/L. This suggests that both Ca complexation and less than optimal precision of the Koukou et al. (2022b) TTT model at low pH and high concentrations of CAC and LAC contributed to the poor performance of this model for dairy foods within their full range of environmental factors (Dataset extremities). The results obtained support that Ca form complexes with CAC and LAC, thereby reducing their growth-inhibitory effects (Wood et al., 1975; Graham and Lund, 1986; Muller et al., 1998). Clearly, this effect of Ca must be quantitatively assessed when recipes preventing growth and toxin formation by proteolytic *C. botulinum* are formulated for Ca-rich dairy products. This effect may also be important for Ca-fortified dairy analogues, and products like Ca-set tofu although not considered as part of the present study.

We have found no previous studies attempting to quantify the effect of Ca concentrations on inhibition of proteolytic *C. botulinum* by CAC and LAC in dairy foods. The present study included the effect of product pH on Ca-acid complexation but effects of storage temperature and of ions such as phosphate melting salts were not evaluated, although potentially relevant (Hacht, 2007; Vavrusova et al., 2014). Challenge tests where TTT were determined did not report measured Ca concentrations in products. Ca concentrations therefore had to be estimated from ingredients in recipes and from Ca content of similar products (Supplementary Table S2). Several assumptions were required to estimate how much Ca concentrations reduce the growth inhibiting effect of CAC and LAC in dairy foods. It is therefore interesting to evaluate if simpler models can predict growth and TTT responses by proteolytic *C. botulinum* in dairy foods.

##### 3.3.3.3 Evaluation of modified TTT models

The modified Koukou et al. (2022b) TTT model used without a term for the effect of interaction between environmental factors and for the full range of the dairy dataset (Dataset extremities; Table 6), provided 17.9% fail-dangerous and 12.2% fail-safe TTT predictions (*n* = 312). B_f-TTT_−/A_f-TTT_-values were 0.92/5.32 (*n*_f_ = 146). Using the same optimized RoA as determined in section 3.3.3.2, this modified Koukou et al. (2022b) TTT model provided two (1.4%) fail-dangerous and 36 (25%) fail-safe TTT predictions (*n* = 144). B_f-TTT_- /A_f-TTT_-values were 0.44/4.5 (*n*_f_ = 100; Table 6).

When the Koukou et al. (2022b) TTT model was modified by replacing terms for undissociated CAC and LAC with terms for total CAC and total LAC it then provided zero (0.0%) fail-dangerous and 23.6% fail-safe TTT predictions for the optimized RoA as determined in section 3.3.3.2 (*n* = 144). B_f-TTT_−/A_f-TTT_-values were 0.37/4.4 (*n*_f_ = 102; Table 6).

**Figure 2:**
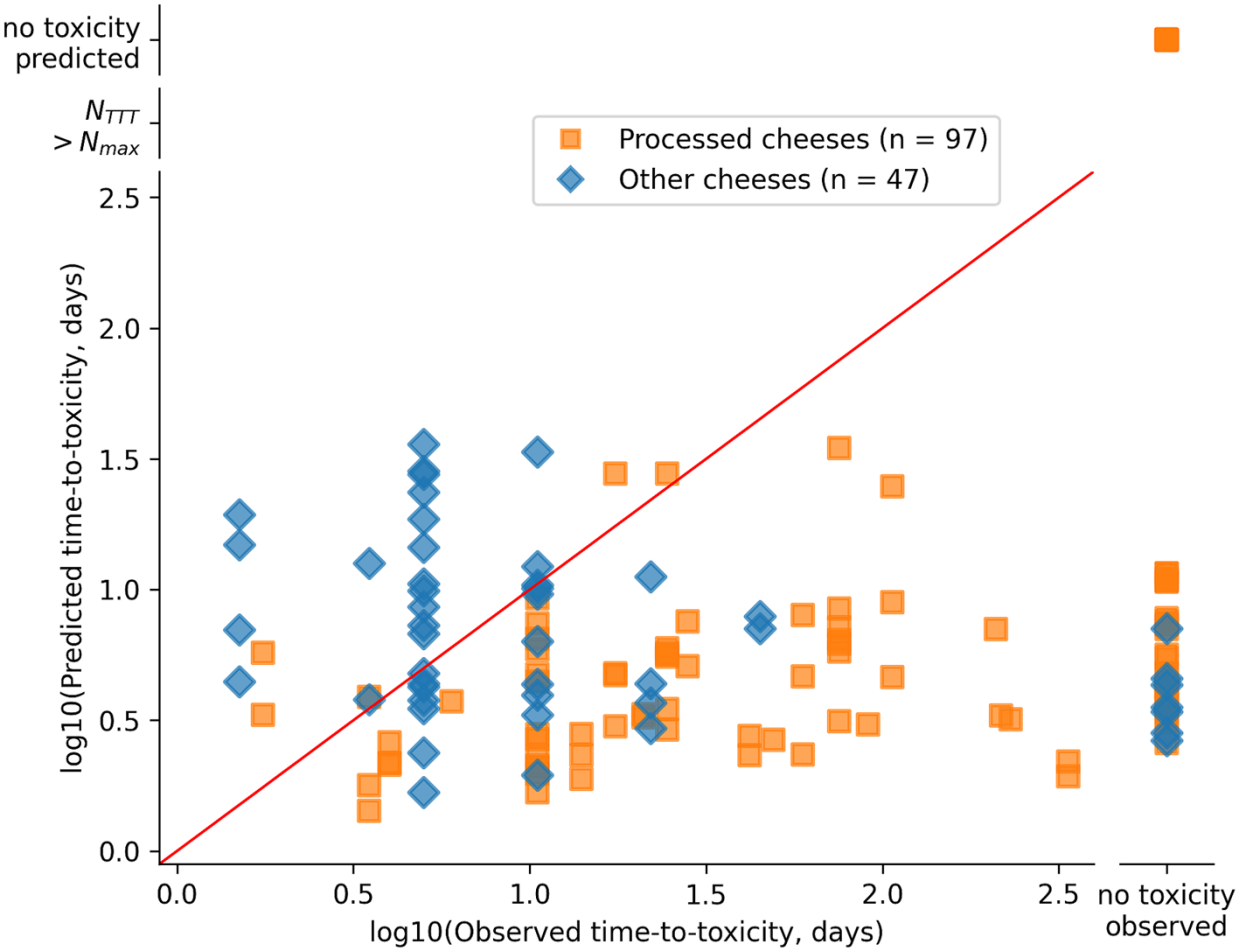
Observed and predicted time-to-toxicity (TTT) for proteolytic *C. botulinum* in challenge tests with processed cheese and other cheeses. TTT was predicted for the optimized range of applicability using the Koukou et al. (2022b) model with total concentrations of citric and lactic acids (see Table 6). Numeric observations on the “no toxicity predicted” axis correspond to fail-dangerous predictions. Points on the no toxicity observed/predicted axis intersections correspond to correct predictions. Conditions in which the predicted population density at the observed time of toxin formation corresponded to N_max_ are shown on the N_TTT_ > N_max_ axis as no quantitatively predicted time for toxin formation was obtained.

The modified Koukou et al. (2022b) TTT model without a term for the effect of interaction between environmental factors had the highest percentage of fail-safe predictions (Tables 5, 6). This was expected as previously observed when cardinal parameter growth models, for different pathogens, were compared with and without terms for the effect of interaction between environmental factors (Mejlholm and Dalgaard, 2009; Martinez-Rios et al., 2025). Similar performance was determined for the Koukou et al. (2022b) TTT model used with Ca complexation and for the simpler and modified Koukou et al. (2022b) TTT model with terms for total CAC and total LAC (Tables 5, 6). The simpler model with total concentrations of CAC and LAC was selected for application with dairy foods (Section 3.4). This model can be used to predict combinations of environmental factors that prevent growth and toxin formation by proteolytic *C. botulinum* in dairy foods. At this stage the model cannot be used for a more accurate prediction of TTT. This is due to variability of TTT responses as shown in Figure 2 and by high A_f-TTT_-values of 6.05 and 2.72 for, respectively, processed cheese and other cheeses (Table 6). This considerable variability between observed and predicted TTT reflected a marked underestimation of TTT for processed cheese by the applied modified Koukou et al. (2022b) TTT model (B_f-TTT_ = 0.18). To evaluate variability between observed and predicted TTT without this bias, the modified Koukou et al. (2022b) TTT model was used for, respectively, processed cheese and other cheeses with *μ_ref_*-values resulting in B_f-TTT_ = 1.00 for both product sub-groups. This provided A_f-TTT_ of 2.66 (*n*_f_ = 102) for dairy foods. A_f-TTT_/*μ_ref_*-values (1/h) were 2.60/0.45 for processed cheese and 2.74/2.80 for other cheeses (Results not shown). Even for product sub-group specific TTT-models, with calibrated *μ_ref_*-values, variability between observed and predicted TTT was larger for dairy foods (A_f-TTT_ of 2.66) compared to non-dairy foods (A_f-TTT_ of 1.93). Various factors most likely contribute to this difference. The present study suggests variation in Ca content of dairy foods and Ca binding/complexation with CAC and LAC are part of those factors. The studied Ca concentrations in products were 4,488 ± 423 mg/kg (AVG ± SD) for processed cheeses and the corresponding values for other cheeses were 2,940 ± 0 mg/kg. Furthermore, some studies of toxin formation in processed cheese used very large time intervals, of up to about 100 days, between some samplings and these experimental designs also contributed to the observed variability (Glass et al., 2017; Supplementary Tables 1, 2). As for non-dairy foods (Section 3.3.2), further research, including studies of well characterized dairy products, seems required before validated models to accurately predict TTT can be developed for dairy foods.

Recently, cardinal parameter growth and growth boundary models for *B. cereus* in dairy foods were developed using a model term for the total concentration of CAC (Maktabdar et al., 2025a,b). In this way, they obtained unbiased predictions and avoided fail-dangerous predictions of growth/no-growth responses specifically related to CAC. However, Maktabdar et al. (2025a) used a dairy substrate (Whey UF permeate solution) to estimate cardinal parameter values for CAC and LAC and the obtained MIC-values were higher than those obtained using a standard laboratory broth (BHI) at the same pH of 6.0. The modified Koukou et al. (2022b) TTT model included MIC-values determined using a liquid laboratory broth (tryptone-peptone-glucose-yeast extract) at 25 °C and pH 6.0. Koukou et al. (2022b) reported the obtained MIC-values in mM for undissociated CAC and LAC. At pH-values below 6.0 the original Koukou et al. (2022b) model relying on concentrations of undissociated CAC and LAC (and used in the present study for non-dairy foods) predicts markedly slower growth than the new modified Koukou et al. (2022b) TTT model with terms for total CAC and total LAC. The reason simply being that concentrations of undissociated acids increase with a factor of about two when pH is decreased by 0.3 units. In contrast, total concentrations of acids remain unchanged at different pH values.

### 3.4 Application of best performing models within their RoA

Most recent UK cases of botulinum for chilled foods were due to proteolytic *C. botulinum*. These products most likely were above 10 °C for a considerable part of their storage time and management of proteolytic *C. botulinum* should include this challenge when processing and product characteristics are designed to prevent growth and toxin formation at likely storage conditions (Anderson et al., 2011; Advisory Committee on the Microbiological Safety of Food (ACMSF), 2023).

The present study used available TTT data to evaluate performance of the Koukou et al. (2022b) growth and growth boundary model for proteolytic *C. botulinum*. Two separate RoAs were determined with respect to product types, product characteristics and storage conditions for (i) the original growth boundary model and non-dairy foods (Section 3.3.2) and (ii) a modified Koukou et al. (2022b) growth boundary model and dairy foods (Section 3.3.3.3). Within their RoAs both models can be used to facilitate identification of conditions that prevent growth and toxin formation by proteolytic *C. botulinum* depending on the combined effect of temperature, storage in air, VP or MAP, pH, WPS, up to four or five organic acids and one or up to three phosphates salts. This is relevant to formulate or re-formulate foods recipes where a pH below 4.6 or above 10% WPS is not compatible with sensory or technological product properties. Due to inherent variability of food product characteristics and less than optimal precision of the models, the identified conditions should not be close to the growth boundary (*ψ* = 1.0) but well into the no-growth region corresponding to a ψ-value of 2.0 or above as previously suggested for both *C. botulinum* and other foodborne pathogens (Mejlholm and Dalgaard, 2009; Koukou et al., 2022a, Maktabdar et al., 2025b). Importantly, the best performing versions of the Koukou et al. (2022b) model, as evaluated and validated in the present study, can predict conditions that prevent growth and toxin formation by proteolytic *C. botulinum* in different types of foods. At his stage, these models are not yet validated to predict TTT for different sub-groups of non-dairy or dairy foods (Sections 3.3.2 and 3.3.3.3.).

Numerous studies of proteolytic *C. botulinum*, particularly in meat and poultry products, focused on the inhibiting effect of curing salts including nitrite, nitrate and ascorbate/iso-ascorbate (Buege et al., 1980; Roberts et al., 1981, 1982; Shakil et al., 2022). However, nitrite added to food has been associated with negative human health effects (Chazelas et al., 2022; Guéraud et al., 2023) and since the 1970’s there have been initiatives to prevent or reduce nitrite in meat products (Buege et al., 1980; Shakil et al., 2022). Recently, EU limited nitrite in meat products to 120 mg NaNO_2_/kg [EU (European Commission), 2023] and France has initiated an action plan to phase out nitrites and nitrates [ANSES (Agence nationale de sécurité sanitair), 2022; Scippo et al., 2025]. The studied versions of the Koukou et al. (2022b) model do not contain the effect of nitrite or nitrate but focuses on combined inhibitory effects including organic acids and this can be most relevant for, e.g., sodium and/or nitrite reduced recipes (Table 8). Scenario 1 and 2 in Table 8 show how predicted combinations of AAC, CAC and LAC can prevent growth (*ψ*-values of 2.1 and 2.2) in a product with pH 5.6, 2.5% WPS and stored at, respectively 25 °C or 15 °C. Importantly, these conditions should be used for products that are heated sufficiently to inactivate psychrotolerant non-proteolytic *C. botulinum* (Group II), for example 90 °C in 10 min, as psychrotolerant *C. botulinum* are predicted to grow at conditions for these two scenarios (Table 8). With the same pH and WPS, scenario 3 and 4 shows how SAC can replace growth inhibition by AAC and LAC and that the inhibiting effect of SAC is markedly reduced in products with higher lipid content (Table 8). Scenarios 5 and 6 show other combinations of pH, WPS and organic acids that are predicted to efficiently prevent growth of proteolytic and non-proteolytic *C. botulinum* in non-dairy foods (Table 8). Several models are available to predict growth or toxin formation by proteolytic *C. botulinum.* Models that exclusively include the effect of temperature, pH and WPS/a_w_ have limited ability to identify combinations of product characteristics and storage conditions that prevent growth and toxin formation. When using exclusively the temperature, pH and WPS/a_w_ of scenarios 1 to 6 in Table 8, models from Combase^2^ and Pathogen Modeling Program (PMP)^3^ predicted growth or toxin formation. However, these predicted responses did not differ markedly from predictions by the Koukou et al. (2022b) model at the same temperature, pH and WPS conditions (Results not shown). The Koukou et al. (2022b) growth and growth boundary model primarily differs from simpler models by including the effect of more environmental factors and the effect of interaction between these factors. These are the key properties that allow versions of the extensive Koukou et al. (2022b) model to predict combination of product characteristics that prevent growth and toxin formation by proteolytic *C. botulinum* (Table 8).

**Table 8:** Predicted combinations of product characteristics and storage temperatures to prevent growth and toxin formation by proteolytic Clostridium botulium in different foods.

Type of foods	Non-diary						Dairy	
Scenarios	1	2	3	4	5	6	7	8
Product characteristics								
Storage temperature, °C	25	15	25	25	15	25	25	25
pH	5.60	5.60	5.60	5.60	6.30	5.40	5.60	5.60
Water phase NaCl, %	2.50	2.50	2.50	2.50	7.00	2.50	1.80	1.80
Acetic acid, mg/L	2,000	1,000	0	2,000	0	3,500	1,200	1,200
Citric acid, mg/L	1,200	1,000	1,000	1,000	1,000	0	5,400	2,500
Lactic acid, mg/L	10,500	7,000	0	7,000	0	7,000	11,000	8,000
Ortho-phosphate ion (P1), %	0.00	0.00	0.00	0.00	0.00	0.00	2.00	2.00
Dry matter in food, % (w/w incl. Lipid)	25	25	25	25	25	25	50	50
Lipid in product, % (w/w)	4	4	4	10	4	4	20	20
Benzoic acid in product, mg/kg	0	0	800	800	800	0	0	0
Sorbic acid in product, mg/kg	0	0	0	0	800	0	0	1,500
Proteolytic *C. botulinum*^a^								
μ_max_, d^−1^	0.00	0.00	0.00	0.00	0.00	0.00	0.00	0.00
Ψ-value	2.07	2.20	>10	3.67	2.18	2.14	2.01	2.27
Non-proteolytic *C. botulinum*^b^								
μ_max_, d^−1^	0.10	0.13	0.00	0.00	0.00	0.00	-^b^	-^b^
Ψ-value	0.92	0.86	>10	1.75	>10	>10	-^b^	-^b^

a Koukou et al. (2022b) growth and growth boundary model for non-dairy foods and modified Koukou et al. (2022b) model including the effect of total rather than undissociated concentrations of citric and lactic acids for dairy foods (See sections 2.4.2 and 3.3.3).

b Koukou et al. (2021) growth and growth boundary model was used for non-dairy foods. In validation studies this model provided 1.1% fail dangerous TTT predictions for meat, poultry, seafood and vegetables but the model has not been evaluated for dairy foods (Koukou et al., 2022a).

Other extensive models have been developed for probability (%) of toxin formation by proteolytic *C. botulinum* in pork slurry after 6 months (Roberts et al., 1981, 1982; Robinson et al., 1982). These models include the effect of temperature, pH, WPS, initial spore concentration, different heat treatments prior to storage, curing salts (initial nitrite and nitrate concentrations, iso-ascorbate and polyphosphate) and SAC. Those extensive probability models, like versions of the Koukou et al. (2022b) model, can facilitate the identification of conditions to prevent toxin formation by proteolytic *C. botulinum.* The two types of models, however, are markedly different with respect to model structure, predicted responses and RoA. The probability models by Roberts et al. (1981) and Robinson et al. (1982) include high nitrite concentrations (100, 200 or 300 mg/kg) and several factors at just two or three specific levels. These models were therefore not included in the present validation study whereas data from Roberts et al. (1982) with 40 mg/kg of nitrite were included (Table 2; Supplementary Table S1).

Dairy foods in general have excellent safety records with respect to botulism and heat treatments, including ultra-high-temperatures (UHT), equivalent to more than 121 °C during 3 min are extensively used for products to be stored at ambient temperatures (Doyle et al., 2015). Pasteurized and hot filled processed cheese can be stored chilled or at ambient temperature and with a shelf-life of several months (ter Steeg and Cuppers, 1995; Glass et al., 2017). These products may contain 40–50% dry matter and water phase concentrations of AAC, CAC and LAC of up to, respectively, 2,400, 7,500 and 17,000 mg/L (Martinez-Rios et al., 2019; Koukou et al., 2022b). For processed cheese, the modified Koukou et al. (2022b) model, including concentrations of total CAC and total LAC (Section 3.3.3.3) provided predictions as shown in Table 8, scenarios 7 and 8. At 25 °C and with 1.80% WPS, pH 5.60, 2.0% P1 in the water phase and water phase concentrations of 1,200 mg/L for AAC, 5,400 mg/L for CAC and 11,000 mg/L for LAC, no growth (ψ-value of 2.01) was predicted for proteolytic *C. botulinum* (Table 8, scenario 7). If lower concentrations of CAC and LAC are desirable, then this can, for example, be obtained using a recipe with SAC (Table 8, Scenario 8).

The two models validated in the present study to predict combination of product characteristics that prevent growth and toxin formation by proteolytic *C. botulinum* should be used in combination with challenge tests and guidelines. Although the present study is comprehensive, the two models were not studied or not successfully validated for all sub-groups of both non-dairy and dairy foods. This includes chemically acidified or fermented foods with pH below 5.1–5.2 (Tables 4, 6), for example, yoghurt, chemically acidified-, brined- and some cream-cheeses, marinated/pickled vegetables and some marinated seafoods including herring products. Challenge tests have the advantages that the combined inhibiting effect of both well-known and other product characteristics (e.g., herbs, spices, bacteriocns and effect of interactions between factors and groups of microorganisms) on TTT can be determined for different specific foods. The limitations of challenge tests, in addition to demanding laboratory requirements, high costs and long time to complete studies, include difficulties with extrapolation of results, and thereby risk ranking, for specific or separate food matrices with different environmental conditions. The present study showed that many challenge tests to determine TTT have been performed without a detailed characterization of the inhibiting environmental factors in the studied products (Supplementary Tables 1, 2). Predictions by the two models validated in the present study can identify interesting combinations of environmental factors to prevent growth in different types of foods and in this way contribute to a more target and efficient use of challenge testing to document food safety. To efficiently use the validated predictive models and challenge testing to support management of proteolytic *C. botulinum*, for example in relation to safety of new or modified food recipes, determination of relevant inhibiting environmental factors is required. This, concur with use of predictive models for other pathogen/food combination (Mejlholm et al., 2010; Martinez-Rios et al., 2020; Koukou et al., 2021, 2022a; Maktabdar et al., 2025b) and with recent recommendation for challenge testing [EURL-Lm (European Union Reference Laboratory for Listeria monocytogenes), 2026] that also support better product characterization to improve food safety management. To facilitate correct application of the Koukou et al. (2022b) model for non-dairy foods and of the modified Koukou et al. (2022b) model, including concentrations of total CAC and total LAC, for dairy foods the two models have been included in the Food Spoilage and Safety Predictor (FSSP) software.^4^ In FSSP, the models can be used within their, respective, RoAs to predict growth rates and ψ-values and in this way facilitate identification of conditions that prevent growth and toxin formation by proteolytic *C. botulinum*. To avoid incorrect application of these models the FSSP software does not include prediction of lag time or TTT by these two models.

## 4 Conclusion and perspectives

Performance of the Koukou et al. (2022b) model was evaluated using 962 TTT responses determined in challenge tests and extracted from the scientific literature for proteolytic *C. botulinum* in non-dairy and dairy foods. The growth and growth boundary model by Koukou et al. (2022b) could be used inside its RoA and with a ψ-value ≥ 2 to determine combinations of product characteristics and storage temperatures that prevent growth and toxin formation by proteolytic *C. botulinum* in the studied non-dairy products without added *Ca.* To perform similar predictions for dairy products the Koukou et al. (2022b) model had to be modified to either (i) take into account complexation between Ca and CAC or LAC in dairy foods or (ii) used with terms for total CAC and total LAC. The model’s RoA with respect to types of products and ranges of environmental factors were determined. To facilitate correct application of the best performing Koukou et al. (2022b) models for non-dairy and dairy foods, they have been included in the Food Spoilage and Safety Predictor (FSSP) software. Further research, including among other aspects studies of well characterized products, seems required before validated models to accurately predict TTT in different foods can be developed.

## Data Availability

The original contributions presented in the study are included in the article/Supplementary material, further inquiries can be directed to the corresponding author.

## Footnotes

1
https://findit.dtu.dk/

2
www.combase.cc

3
https://pmp.errc.ars.usda.gov/default.aspx

4
fssp.food.dtu.dk

## References

[ref1] Advisory Committee on the Microbiological Safety of Food (ACMSF). (2023). Report on Botulinum Neurotoxin-Producing Clostridia Working Group on Botulinum Neurotoxin-Producing Clostridia. doi: 10.46756/sci.fsa.ozk974

[ref2] Anderson N. M. Larkin J. W. Cole M. B. Skinner G. E. Whiting R. C. Gorris L. G. M. . (2011). Food safety objective approach for controlling *Clostridium botulinum* growth and toxin production in commercially sterile foods. J. Food Prot. 74, 1956–1989. doi: 10.4315/0362-028X.JFP-11-082, 22054200

[ref3] ANSES (Agence nationale de sécurité sanitair) (2022). Opinion of the French Agency for Food, Environmental and Occupational Health & Safety on the Risk Associated with the Consumption of Nitrites and Nitrates. Available online at: https://www.anses.fr/en/content/anses-revised-opinion-risks-associated-consumption-nitrites-and-nitrates (Accessed July 31, 2024).

[ref4] Austin J. W. Dodds K. L. Blanchfield B. Farber J. M. (1998). Growth and toxin production by *Clostridium botulinum* on inoculated fresh-cut packaged vegetables. J. Food Prot. 61, 324–328. doi: 10.4315/0362-028x-61.3.324, 9708304

[ref5] Barbut S. Tanaka N. Gassens R. G. Maurer A. J. (1986). Effects of sodium chloride reduction and polyphosphate addition on *Clostridium botulinum* toxin production in Turkey frankfurters. J. Food Sci. 51, 1136–1172. doi: 10.1111/j.1365-2621.1986.tb13066.x

[ref6] Bonventre P. F. Kempe L. L. (1960). Physiology of toxin production by *Clostridium botulinum* types A and B. J. Bacteriol. 79, 18–23. doi: 10.1128/jb.79.1.18-23.1960, 13802633 PMC278628

[ref7] Briozzo J. Amato de Lagarde E. Chirife J. Parada J. L. (1983). *Clostridium botulinum* type A growth and toxin production in media and process cheese spread. Appl. Environ. Microbiol. 45, 1150–1152. doi: 10.1128/aem.45.3.1150-1152.1983, 6342535 PMC242424

[ref8] Brocklehurst T. F. Wilson P. D. G. (2000). The role of lipids in controlling microbial growth. Grasas Aceites 51, 66–73. doi: 10.3989/gya.2000.v51.i1-2.408

[ref9] Budavari S. (1989). The Merck Index: An Encyclopedia of Chemicals, Drugs and Biologicals. 11th Edn Rahway, NJ: Merck & Co., Inc.

[ref10] Buege D. R. Birdsall J. J. Dryden F. D. Judge M. D. Kemp J. D. Rogers R. W. (1980). Update: Nitrite-free Processed Meats Proceedings of the 33rd Reciprocal Meat Conference, 122–135. Available online at: https://meatscience.org/docs/default-source/publications-resources/rmc/1980/update---nitrite-free-processed-meats.pdf

[ref11] Carter A. T. Peck M. W. (2014). Genomes, neurotoxins and biology of *Clostridium botulinum* group I and group II. Res. Microbiol. 166, 303–317. doi: 10.1016/j.resmic.2014.10.010, 25445012 PMC4430135

[ref12] Chaidoutis E. Keramydas D. Papalexis P. Migdanis A. Migdanis I. Lazaris A. C. . (2022). Foodborne botulism: a brief review of cases transmitted by cheese products (review). Biomed. Rep. 16:41. doi: 10.3892/br.2022.1524, 35386113 PMC8972315

[ref13] Chazelas E. Pierre F. Druesne-Pecollo N. Esseddik Y. Szabo de Edelenyi F. Agaesse C. . (2022). Nitrites and nitrates from food additives and natural sources and cancer risk: results from the NutriNet-Santé cohort. Int. J. Epidemiol. 51, 1106–1119. doi: 10.1093/ije/dyac046, 35303088 PMC9365633

[ref14] Christiansen L. N. Tompkin R. B. Shaparis A. B. Kueper T. V. Johnston R. W. Kautter D. A. . (1974). Effect of sodium nitrite on toxin production by *Clostridium botulinum* in bacon. Appl. Microbiol. 27, 733–737. doi: 10.1128/am.27.4.733-737.1974, 4596753 PMC380125

[ref15] Connan C. Denève C. Mazuet C. Popoff M. R. (2013). Regulation of toxin synthesis in *Clostridium botulinum* and *Clostridium tetani*. Toxicon 75, 90–100. doi: 10.1016/j.toxicon.2013.06.001, 23769754

[ref16] Daifas D. P. Smith J. P. Blanchfield B. Austin J. W. (1999a). Effect of pH and CO_2_ on growth and toxin production by *Clostridium botulinum* in English-style crumpets packaged under modified atmospheres. J. Food Prot. 62, 1157–1161. doi: 10.4315/0362-028x-62.10.1157, 10528719

[ref17] Daifas D. P. Smith J. Blanchfield B. Austin J. W. (1999b). Growth and toxin production by *Clostridium botulinum* in English-style crumpets packaged under modified atmospheres. J. Food Prot. 62, 349–355. doi: 10.4315/0362-028x-62.4.349, 10419207

[ref18] Dalgaard P. Jørgensen L. V. (2000). Cooked and brined shrimps packed in modified atmosphere have a shelf-life of >7 months at 0 °C, but spoil in 4-6 days at 25 °C. Int. J. Food Sci. Technol. 35, 431–442. doi: 10.1046/j.1365-2621.2000.00402.x

[ref19] Dalgaard P. Mejlholm O. (2019). “Modelling growth of *Listeria monocytogenes* and lactic acid bacteria in food environments,” in Foodborne Bacterial Pathogens: Methods and Protocols, Methods in Molecular Biology, ed. Bridier A., vol. 1918 (Springer Science + Business Media), 247–264. 10.1007/978-1-4939-9000-9_20 30580414

[ref20] Dando P. R. (1969). Lactate metabolism in fish. J. Mar. Biol. Assoc. U. K. 49, 209–223. doi: 10.1017/S002531540004652X

[ref21] Deshwal G. K. van der Meulen L. Huppertz T. (2024). Distribution of salts in milk and cheese: critical methodological aspects. Encyclopedia 4, 1629–1643. doi: 10.3390/encyclopedia4040107

[ref22] Dodds K. L. (1989). Combined effect of water activity and pH on inhibition of toxin production by *Clostridium botulinum* in cooked, vacuum-packed potatoes. Appl. Environ. Microbiol. 55, 656–660. doi: 10.1128/aem.55.3.656-660.1989, 2648990 PMC184175

[ref23] Dodds K. L. (1994). “*Clostridium botulinum*,” in Foodborne Disease Handbook. Vol 1. Diseases Caused by Bacteria, eds. Hui Y. H. Gorham J. R. Murrell K. D. Cliver D. O. (New York: Marcel Dekker, Inc.), 97–131.

[ref24] Doyle C. J. Gleeson D. Jordan K. Beresford T. P. Ross R. P. Fitzgerald G. F. . (2015). Anaerobic sporeformers and their significance with respect to milk and dairy products. Int. J. Food Microbiol. 197, 77–87. doi: 10.1016/j.ijfoodmicro.2014.12.022 25574847

[ref25] EU (European Commission) (2023). Commission Regulation (EU) 2023/2108 of 6 October 2023 Amending annex II to Regulation (EC) No 1333/2008 of the European Parliament and of the Council and the annex to Commission Regulation (EU) No 231/2012 as Regards Food Additives Nitrites (E 249–250) and Nitrates (E 251–252). Available online at: https://eur-lex.europa.eu/legal-content/EN/TXT/PDF/?uri=CELEX:32023R2108 (Accessed July 31, 2024).

[ref26] EURL-Lm (European Union Reference Laboratory for *Listeria monocytogenes*) (2026). Technical Guidance Document on challenge Tests and Durability Studies for Assessing shelf-life of Ready-to-eat Foods Related to *Listeria monocytogenes* Version 4 of 1 July 2021, Amendment 1 of 26 February 2026. Available online at: https://zenodo.org/records/18783767 (Accessed May 10, 2026).

[ref27] Fleck-Derderian S. Shankar M. Rao A. K. Chatham-Stephens K. Adjei S. Sobel J. . (2018). The epidemiology of foodborne botulism outbreaks: a systematic review. Clin. Infect. Dis. 66, S73–S81. doi: 10.1093/cid/cix846, 29293934

[ref28] Franciosa G. (2020). What links the mascarpone and nacho cheese sauce outbreaks of botulism. Clin. Infect. Dis. 70:1792. doi: 10.1093/cid/ciz700, 31342063

[ref29] Gibson A. M. Bratchell N. Roberts T. A. (1987). The effect of sodium chloride and temperature on the rate and extent of growth of *Clostridium botulinum* type a in pasteurized pork slurry. J. Appl. Bacteriol. 62, 479–490. doi: 10.1111/j.1365-2672.1987.tb02680.x, 3305458

[ref30] Glass K. A. Doyle M. P. (1991). Relationship between water activity of fresh pasta and toxin production by proteolytic *Clostridium botulinum*. J. Food Prot. 54, 162–165. doi: 10.4315/0362-028X-54.3.162, 31051648

[ref31] Glass K. A. Golden M. C. Wanless B. J. Conklin T. Schweihofer J. P. Schill K. M. (2024). Inhibition of *Listeria monocytogenes* and *Clostridium botulinum* in uncured shredded pork and Turkey packaged under reduced oxygen conditions. J. Food Prot. 87:100271. doi: 10.1016/j.jfp.2024.100271, 38561027

[ref32] Glass K. A. Johnson E. A. (2004a). Factors that contribute to the botulinal safety of reduced-fat and fat-free process cheese products. J. Food Prot. 67, 1687–1693. doi: 10.4315/0362-028x-67.8.1687, 15330535

[ref33] Glass K. A. Johnson E. A. (2004b). Antagonistic effect of fat on the antibotulinal activity of food preservatives and fatty acids. Food Microbiol. 21, 675–682. doi: 10.1016/j.fm.2004.03.002

[ref34] Glass K. A. Mu M. LeVine B. Rossi F. (2017). Inhibition of *Clostridium botulinum* in model reduced-sodium pasteurized prepared cheese products. J. Food Prot. 80, 1478–1488. doi: 10.4315/0362-028X.JFP-17-027, 28786718

[ref35] Golden M. C. Wanless B. J. David J. R. D. Kottapalli B. Lineback D. S. Talley R. J. . (2017). Effect of cultured celery juice, temperature, and product composition on the inhibition of proteolytic *Clostridium botulinum* toxin production. J. Food Prot. 80, 1259–1265. doi: 10.4315/0362-028X.JFP-17-011, 28686493

[ref36] Graham A. F. Lund B. M. (1986). The effect of citric acid on growth of proteolytic strains of *Clostridium botulinum*. J. Appl. Bacteriol. 61, 39–49. doi: 10.1111/j.1365-2672.1986.tb03756.x, 3531139

[ref37] Guéraud F. Buisson C. Promeyrat A. Naud N. Fouché E. Bézirard V. . (2023). Effects of sodium nitrite reduction, removal or replacement on cured and cooked meat for microbiological growth, food safety, colon ecosystem, and colorectal carcinogenesis in Fischer 344 rats. NPJ Sci. Food. 7:53. doi: 10.1038/s41538-023-00228-9, 37805637 PMC10560221

[ref38] Guinee T. P. Feeney E. P. Auty M. a. E. Fox P. F. (2002). Effect of pH and calcium concentration on some textural and functional properties of mozzarella cheese. J. Dairy Sci. 85, 1655–1669. doi: 10.3168/jds.S0022-0302(02)74238-0 12201515

[ref39] Guinee T. P. O’Kennedy B. T. (2009). The effect of calcium content of Cheddar-style cheese on the biochemical and rheological properties of processed cheese. Dairy Sci. Technol. 89, 317–333. doi: 10.1051/dst/2009009

[ref40] Hacht B. (2007). Complex formation of acetic acid with ca(II) and Mg(II) under physiological conditions. J. Solut. Chem. 37, 155–163. doi: 10.1007/s10953-007-9233-3, 30311153

[ref41] Hauschild A. H. W. Hilsheimer R. (1979). Effect of salt content and pH on toxigenesis by *Clostridium botulinum* in caviar. J. Food Prot. 42, 245–248. doi: 10.4315/0362-028X-42.3.245, 30812291

[ref42] Hauschild A. Hilsheimer R. Jarvis G. Raymond D. P. (1982). Contribution of nitrite to the control of *Clostridium botulinum* in liver sausage. J. Food Prot. 45, 500–506. doi: 10.4315/0362-028X-45.6.500, 30866230

[ref43] Hayashi K. Sakaguchi S. Sakaguchi G. (1986). Primary multiplication of *Clostridium botulinum* type A in mustard-miso stuffing of 'karashi-renkon' (deep-fried mustard-stuffed lotus root). Int. J. Food Microbiol. 3, 311–320. doi: 10.1016/0168-1605(86)90013-9

[ref44] Huhtanen C. N. Feinberg J. I. Trenchard H. Phillips J. G. (1983). Acid enhancement of *Clostridium botulinum* inhibition in ham and bacon prepared with potassium sorbate and sorbic acid. J. Food Prot. 46, 807–810. doi: 10.4315/0362-028X-46.9.807, 30921946

[ref45] Hunter J. D. (2007). Matplotlib: a 2D graphics environment. Comput. Sci. Eng. 9, 90–95. doi: 10.1109/MCSE.2007.55

[ref46] International Commission on Microbiological Specifications for Foods (ICMSF) (1996). Microorganisms in Foods 5: Characteristics of Microbial Pathogens. New York, NY: Springer.

[ref47] Ito K. Chen J. Lerke P. Seeger M. L. Unverferth J. A. (1976). Effect of acid and salt concentration in fresh-pack pickles on the growth of *Clostridium botulinum* spores. Appl. Environ. Microbiol. 32, 121–124. doi: 10.1128/aem.32.1.121-124.1976, 9898 PMC170016

[ref48] Ito K. A. Chen J. K. Seeger M. L. Unverferth J. A. Kimball R. N. (1978). Effect of pH on the growth of *Clostridium botulinum* in canned figs. J. Food Sci. 43, 1634–1635. doi: 10.1111/j.1365-2621.1978.tb02564.x

[ref49] Ivey F. J. Robach M. C. (1978). Effect of sorbic acid and sodium nitrite on *Clostridium botulinum* outgrowth and toxin production in canned comminuted pork. J. Food Sci. 43, 1782–1785. doi: 10.1111/j.1365-2621.1978.tb07413.x

[ref50] Ivey F. J. Shaver K. J. Christiansen L. N. Tompkin R. B. (1978). Effect of potassium sorbate on toxinogenesis by *Clostridium botulinum* in bacon. J. Food Prot. 41, 621–625. doi: 10.4315/0362-028X-41.8.621, 30795120

[ref51] Jiang Y. Barone G. Rauh V. Lillevang S. K. Skibsted L. H. Ahrné L. (2023). Temperature effects on calcium partition kinetics in pasteurised skim milk during storage. Int. Dairy J. 137:105518. doi: 10.1016/j.idairyj.2022.105518

[ref52] Jun J. Jung M. Kim D. Jeong I. Kim B. (2017). Postmortem changes in physiochemical and sensory properties of red snow crab (*Chionoecetes japonicus*) leg muscle during freeze storage. Fish. Aquat. Sci. 20:13. doi: 10.1186/s41240-017-0057-9

[ref53] Juneja V. K. Marks H. M. (1999). Proteolytic *Clostridium botulinum* growth at 12–48°C simulating the cooling of cooked meat: development of a predictive model. Food Microbiol. 16, 583–592. doi: 10.1006/fmic.1999.0270

[ref54] Juneja V. K. Purohit A. S. Golden M. C. Osoria M. Glass A. K. Mishra A. . (2021a). A predictive growth model for *Clostridium botulinum* during cooling of cooked uncured ground beef. Food Microbiol. 93:103618. doi: 10.1016/j.fm.2020.103618, 32912576

[ref55] Juneja V. K. Sidhub G. Xu X. Osoria M. Glass A. K. Schill K. M. . (2022). Predictive model for growth of *Clostridium botulinum* from spores at temperatures applicable to cooling of cooked ground pork. Innov. Food Sci. Emerg. Technol. 77:102960. doi: 10.1016/j.ifset.2022.102960

[ref56] Juneja V. K. Xu X. Osoria M. Glass A. K. Schill K. M. Golden M. C. . (2021b). Predictive model for growth of *Clostridium botulinum* from spores during cooling of cooked ground chicken. Food Res. Int. 149:110695. doi: 10.1016/j.foodres.2021.110695, 34600690

[ref57] Karahadian C. Lindsay R. C. (1987). Integrated roles of lactate, ammonia, and calcium in texture development of mold surface-ripened cheese. J. Dairy Sci. 70, 909–918. doi: 10.3168/jds.S0022-0302(87)80094-2

[ref58] Kasai Y. Kimura B. Kawasaki S. Fukaya T. Sakuma K. Fujii T. (2005). Growth and toxin production by *Clostridium botulinum* in steamed rice aseptically packed under modified atmosphere. J. Food Prot. 68, 1005–1011. doi: 10.4315/0362-028X-68.5.1005, 15895734

[ref59] Kaufmann O. W. Marshall R. S. (1965). Factors affecting the development of *Clostridium botulinum* in whole milk. Appl. Microbiol. 13, 521–526. doi: 10.1128/am.13.4.521-526.1965, 14339256 PMC1058291

[ref60] Kim D. H. Seong P. N. Cho S. H. Kim J. H. Lee J. M. Jo C. . (2016). Pork quality traits according to postmortem pH and temperature in Berkshire. Asian Australas. J. Anim. Sci. 29, 1338–1344. doi: 10.5851/kosfa.2016.36.1.29 27499661 PMC4973946

[ref61] Kimura B. Kimura R. Fukaya T. Sakuma K. Miya S. Fujii T. (2008). Growth and toxin production of proteolytic *Clostridium botulinum* in aseptically steamed rice products at pH 4.6 to 6.8, packed under modified atmosphere, using a deoxidant pack. J. Food Prot. 71, 468–472. doi: 10.4315/0362-028X-71.3.468, 18389687

[ref62] Koukou I. Devitt T. D. Dalgaard P. (2022a). Extensive growth and growth boundary model for non-proteolytic *Clostridium botulinum* – evaluation and validation with MAP and smoked foods. Food Microbiol. 102:103931. doi: 10.1016/j.fm.2021.103931 34809957

[ref63] Koukou I. Mejlholm O. Dalgaard P. (2021). Cardinal parameter growth and growth boundary model for non-proteolytic *Clostridium botulinum* – effect of eight environmental factors. Int. J. Food Microbiol. 346:109162. doi: 10.1016/j.ijfoodmicro.2021.109162, 33827003

[ref64] Koukou I. Stergioti T. la Cour R. Gkogka E. Dalgaard P. (2022b). *Clostridium sporogenes* as surrogate for proteolytic *C. botulinum* - development and validation of extensive growth and growth-boundary model. Food Microbiol. 107:104060. doi: 10.1016/j.fm.2022.104060, 35953193

[ref65] Lalitha K. V. Gopakumar K. (2001). Growth and toxin production by *Clostridium botulinum* in fish (*Mugil cephalus*) and shrimp (*Penaeus indicus*) tissue homogenates stored under vacuum. Food Microbiol. 18, 651–657. doi: 10.1006/fmic.2001.0433

[ref66] Lambert A. D. Smith J. P. Dodds K. L. (1991). Effect of headspace C0_2_ concentration on toxin production by *Clostridium botulinum* in MAP, irradiated fresh pork. J. Food Prot. 54, 588–592. doi: 10.4315/0362-028X-54.8.588, 31051603

[ref67] Larson A. E. Johnson E. A. Barmore C. R. Hughes M. D. (1997). Evaluation of the botulism hazard from vegetables in modified atmosphere packaging. J. Food Prot. 60, 1208–1214. doi: 10.4315/0362-028X-60.10.1208, 31207733

[ref68] Le Marc Y. Huchet V. Bourgeois C. M. Guyonnet J. P. Mafart P. Thuault D. (2002). Modelling the growth kinetics of *Listeria* as a function of temperature, pH and organic acid concentration. Int. J. Food Microbiol. 73, 219–237. doi: 10.1016/S0168-1605(01)00640-7, 11934031

[ref69] Li H. Guo Y. Tian T. Guo W. Liu C. Liang X. . (2022). Epidemiological analysis of foodborne botulism outbreaks — China, 2004–2020. China CDC Wkly 4, 788–792. doi: 10.46234/ccdcw2022.114, 36284604 PMC9547723

[ref70] Lindström M. Myllykoski J. Sivelä S. Korkeala H. (2010). *Clostridium botulinum* in cattle and dairy products. Crit. Rev. Food Sci. Nutr. 50, 281–304. doi: 10.1080/10408390802544405, 20301016

[ref71] Liu X.-C. Kirkensgaard J. J. K. Skibsted L. H. (2021). Temperature effects on spontaneous supersaturation of calcium citrate in presence of lactate. Int. Dairy J. 118:105023. doi: 10.1016/j.idairyj.2021.105023

[ref72] Lövenklev M. Holst E. Borch E. Rådström P. (2004). Relative neurotoxin gene expression in *Clostridium botulinum* type B, determined using quantitative reverse transcription-PCR. Appl. Environ. Microbiol. 70, 2919–2927. doi: 10.1128/aem.70.5.2919-2927.2004, 15128552 PMC404387

[ref73] Lu Z. Fleming H. P. McFeeters R. F. (2002). Effects of fruit size on fresh cucumber composition and the chemical and physical consequences of fermentation. J. Food Sci. 67, 2934–2939. doi: 10.1111/j.1365-2621.2002.tb08841.x

[ref74] Lubin L. B. Morton R. D. Bernard D. T. (1985). Toxin production in hard-cooked eggs experimentally inoculated with *Clostridium botulinum*. J. Food Sci. 50, 969–984. doi: 10.1111/j.1365-2621.1985.tb12991.x

[ref75] Lund B. M. Graham A. F. George S. M. (1988). Growth and formation of toxin by *Clostridium botulinum* in peeled, inoculated, vacuum-packed potatoes after a double pasteurization and storage at 25 °C. J. Appl. Bacteriol. 64, 241–246. doi: 10.1111/j.1365-2672.1988.tb03381.x, 3290178

[ref76] Lúquez C. Edwards L. Griffin C. Sobel J. (2021). Foodborne botulism outbreaks in the United States, 2001–2017. Front. Microbiol. 12:713101. doi: 10.3389/fmicb.2021.713101, 34335550 PMC8322756

[ref77] Maas M. R. Glass K. A. Doyle M. P. (1989). Sodium lactate delays toxin production by *Clostridium botulinum* in cook-in-bag Turkey products. Appl. Environ. Microbiol. 55, 2226–2229. doi: 10.1128/aem.55.9.2226-2229.1989, 2679382 PMC203060

[ref78] Maktabdar M. Houmann R. H. Scheel N. H. Skytthe K. B. Wemmenhove E. Gkogka E. . (2025b). Evaluation and validation of extensive growth and growth boundary models for mesophilic and psychrotolerant *Bacillus cereus* in dairy products (part 2). Front. Microbiol. 16:553903. doi: 10.3389/fmicb.2025.1553903, 40231235 PMC11994723

[ref79] Maktabdar M. Wemmenhove E. Gkogka E. Dalgaard P. (2025a). Development of extensive growth and growth boundary models for mesophilic and psychrotolerant *Bacillus cereus* in dairy products (part 1). Front. Microbiol. 16:1553885. doi: 10.3389/fmicb.2025.1553885, 40190734 PMC11968683

[ref80] Martinez-Rios V. Gkogka E. Dalgaard P. (2020). Predicting growth of *Listeria monocytogenes* at dynamic conditions during manufacturing, ripening and storage of cheeses – evaluation and application of models. Food Microbiol. 92:103578. doi: 10.1016/j.fm.2020.103578, 32950162

[ref81] Martinez-Rios V. Idrizi R. Dalgaard P. Hansen L. T. Hansen T. B. (2025). Modelling and predicting growth and growth boundary of *Bacillus cereus* s.l. from phylogroups II, IV, V and VI in starchy foods at or below 12 °C. Front. Microbiol. 16:1531014. doi: 10.3389/fmicb.2025.1531014, 40376460 PMC12080233

[ref82] Martinez-Rios V. Jørgensen M. Ø. Koukou I. Gkogka E. Dalgaard P. (2019). Growth and growth boundary model with terms for melting salts to predict growth responses of *Listeria monocytogenes* in spreadable processed cheese. Food Microbiol. 84:103255. doi: 10.1016/j.fm.2019.103255, 31421751

[ref83] Matsumoto M. Yamanaka H. (1990). Post-mortem biochemical changes in the muscle of kuruma prawn during storage and evaluation of the freshness. Nippon Suisan Gakkaishi 56, 1145–1149. doi: 10.2331/suisan.56.1145

[ref84] Mejlholm O. Bøknæs N. Dalgaard P. (2015). Development and validation of a stochastic model for potential growth of *Listeria monocytogenes* in naturally contaminated lightly preserved seafood. Food Microbiol. 45, 276–289. doi: 10.1016/j.fm.2014.06.006, 25500393

[ref85] Mejlholm O. Dalgaard P. (2009). Development and validation of an extensive growth and growth boundary model for *Listeria monocytogenes* in lightly preserved and ready-to-eat shrimp. J. Food Prot. 72, 2132–2143. doi: 10.4315/0362-028X-72.10.2132, 19833037

[ref86] Mejlholm O. Gunvig A. Borggaard C. Blom-Hanssen J. Mellefont L. Ross T. . (2010). Predicting growth rates and growth boundary of *Listeria monocytogenes* - an international validation study with focus on processed and ready-to-eat meat and seafood. Int. J. Food Microbiol. 141, 137–150. doi: 10.1016/j.ijfoodmicro.2010.04.026, 20570006

[ref87] Meng J. Genigeorgis C. A. (1994). Delaying toxigenesis of *Clostridium botulinum* by sodium lactate in ‘sous-vide’ products. Lett. Appl. Microbiol. 19, 20–23. doi: 10.1111/j.1472-765X.1994.tb00893.x

[ref88] Merialdi G. Ramini M. Parolari G. Barbuti S. Frustoli M. A. Taddei R. . (2016). Study on potential *Clostridium botulinum* growth and toxin production in Parma ham. J. Food Saf. 5:5564. doi: 10.4081/ijfs.2016.5564, 27800441 PMC5076734

[ref89] Miller A. J. Call J. E. Whiting R. C. (1993). Comparison of organic acids for *Clostridium botulinum* control in an uncured Turkey product. J. Food Prot. 56, 958–962. doi: 10.4315/0362-028X-56.11.958 31113090

[ref90] Muller E. Al-Attar J. Wolff A. G. Farber B. F. (1998). Mechanism of salicylate-mediated inhibition of biofilm in *Staphylococcus epidermidis*. J. Infect. Dis. 177, 501–503. doi: 10.1086/517386, 9466548

[ref91] Nelson K. A. Busta F. F. Sofos J. N. Wagner M. K. (1983). Effect of polyphosphates in combination with nitrite-sorbate or sorbate on *Clostridium botulinum* growth and toxin production in chicken frankfurter emulsions. J. Food Prot. 46, 846–851. doi: 10.4315/0362-028X-46.10.846, 30921838

[ref92] Pelroy G. A. Eklund M. W. Paranjpye R. N. Suzuki E. M. Peterson M. E. (1982). Inhibition of *Clostridium botulinum* types A and E toxin formation by sodium nitrite and sodium chloride in hot-process (smoked) Salmon. J. Food Prot. 45, 833–841. doi: 10.4315/0362-028X-45.9.833, 30866305

[ref93] Pereira J. P. F. Mageste A. C. Da Silva Campos N. De Sousa R. A. Francisquini J. D. Perrone Í. T. . (2019). Calcium partition in Minas Padrão cheese and its bioaccessibility during ripening time. Food Sci. Technol. 39, 859–866. doi: 10.1590/fst.12518

[ref94] Pernu N. Keto-Timonen R. Lindström M. Korkeala H. (2020). High prevalence of *Clostridium botulinum* in vegetarian sausages. Food Microbiol. 91:103512. doi: 10.1016/j.fm.2020.103512, 32539985

[ref95] Puolanne E. Pösö A. R. Ruusunen M. Sepponen K. Kylä-Puhju M. (2002). Lactic Acid in Muscle and its Effects on Meat Quality Proceedings of the 55th Reciprocal Meat Conference, vol. 55 American Meat Science Association, 57–62.

[ref96] Python Software Foundation (2021). Python 3.10.0. Available online at: https://www.python.org/downloads/release/python-3100/ (Accessed May 10, 2026).

[ref97] Rawson A. M. Dempster A. W. Humphreys Minton N. P. (2023). Pathogenicity and virulence of *Clostridium botulinum*. Virulence 14:2205251. doi: 10.1080/21505594.2023.2205251 37157163 PMC10171130

[ref98] RIVM (2025). NEVO online versie 2025/9.0. Available online at: https://www.rivm.nl/nederlands-voedingsstoffenbestand (Accessed April 5, 2026).

[ref99] Roberts T. A. Gibson A. M. Robinson A. (1981). Prediction of toxin production by *Clostridium botulinum* in pasteurized pork slurry. Int. J. Food Sci. Technol. 16, 337–355. doi: 10.1111/j.1365-2621.1981.tb01827.x

[ref100] Roberts T. A. Gibson A. M. Robinson A. (1982). Factors controlling the growth of *Clostridium botulinum* types a and B in pasteurized, cured meats. III. The effect of potassium sorbate. Int. J. Food Sci. Technol. 17, 307–326. doi: 10.1111/j.1365-2621.1982.tb00187.x

[ref101] Robinson A. Gibson A. M. Roberts T. A. (1982). Factors controlling the growth of *Clostridium botulinum* types a and B in pasteurized, cured meats. V. Prediction of toxin production. Non-linear effects of storage temperature and salt concentration. Int. J. Food Sci. Technol. 17, 727–744. doi: 10.1111/j.1365-2621.1982.tb00233.x

[ref102] Ross T. (1996). Indices for performance evaluation of predictive models in food microbiology. J. Appl. Bacteriol. 81, 501–508. doi: 10.1111/j.1365-2672.1996.tb03539.x, 8939028

[ref103] Ross T. (1999). Predictive Food Microbiology Models in the Meat Industry. Sydney: Meat and Livestock Australia, 196.

[ref104] Ross T. Baranyi J. McMeekin T. A. (2000). “Predictive microbiology and food safety,” in Encyclopaedia of Food Microbiology, eds. Robinson R. Batt C. A. Patel P. (London: Academic Press), 1699–1710.

[ref105] Ross T. Dalgaard P. (2004). “Secondary models,” in Modeling Microbial Responses in Foods, eds. McKellar R. C. Lu X. (Boca Raton, FL: CRC Press), 63–150.

[ref106] Saleh M. A. Ordal J. (1955). Studies on growth and toxin production of *Clostridium botulinum* in a precooked frozen food. II. Inhibition by lactic acid bacteria. Food Res. 20, 340–350. doi: 10.1111/j.1365-2621.1955.tb16847.x

[ref107] Schaffner D. W. Ross W. H. Montville T. J. (1998). Analysis of the influence of environmental parameters on *Clostridium botulinum* time-to-toxicity by using three modeling approaches. Appl. Environ. Microbiol. 64, 4416–4422. doi: 10.1128/AEM.64.11.4416-4422.1998, 9797300 PMC106662

[ref108] Scippo M. Badot P. Bornert G. Desriac N. Dubois-Brissonnet F. Gutierrez A. E. . (2025). Opinion of the French Agency for Food, environmental and Occupational Health & Safety (ANSES) on the risks associated with the consumption of nitrites and nitrates. Food Risk Assess Europe 3. doi: 10.2903/fr.efsa.2025.fr-0059

[ref109] Shakil M. H. Trisha A. T. Rahman M. Talukdar S. Kobun R. Huda N. . (2022). Nitrites in cured meats, health risk issues, alternatives to nitrites: a review. Foods 11:3355. doi: 10.3390/foods11213355, 36359973 PMC9654915

[ref110] Shkembi B. Huppertz T. (2022). Calcium absorption from food products: food matrix effects. Nutrients 14. doi: 10.3390/nu14010180, 35011055 PMC8746734

[ref111] Showkat Q. A. Majid D. Makroo H. Dar B. (2021). Physico-mechanical characterization of different grades of Lotus rhizome (*Nelumbo nucifera* Gaertn) for valorisation and smart post-harvest management. Appl. Food Res. 1:100002. doi: 10.1016/j.afres.2021.100002

[ref112] Shumilina E. Ciampa A. Capozzi F. Rustad T. Dikiy A. (2015). NMR approach for monitoring post-mortem changes in Atlantic salmon fillets stored at 0 and 4°C. Food Chem. 184, 12–22. doi: 10.1016/j.foodchem.2015.03.037, 25872421

[ref113] Simpson M. V. Smith J. P. Dodds K. Ramaswamy H. S. Blanchfield B. Simpson. (1995). Challenge studies with *Clostridium botulinum* in a sous-vide spaghetti and meat-sauce product. J. Food Prot. 58, 229–234. doi: 10.4315/0362-028X-58.3.229 31137299

[ref114] Skinner G. E. Solomon H. M. Fingerhut G. A. (1999). Prevention of *Clostridium botulinum* type a proteolytic B and E toxin formation in refrigerated pea soup by *Lactobacillus plantarum* ATCC 8014. J. Food Sci. 64, 724–727. doi: 10.1111/j.1365-2621.1999.tb15119.x

[ref115] Smith D. P. Fletcher D. L. Papa C. M. (1992). Post-mortem biochemistry of Pekin duckling and broiler chicken pectoralis muscle. Poultry Sci. 71, 1768–1772. doi: 10.3382/ps.0711768, 1454694

[ref116] Solomon H. M. Kautter D. A. (1986). Growth and toxin production by *Clostridium botulinum* in sauteed onions. J. Food Prot. 49, 618–620. doi: 10.4315/0362-028X-49.8.618, 30959688

[ref117] Solomon H. M. Lynt R. K. Lilly T. Kautter D. A. (1977). Effect of low temperatures on growth of *Clostridium botulinum* spores in neat of the blue crab. J. Food Prot. 40, 5–7. doi: 10.4315/0362-028X-40.1.5, 30731557

[ref118] Solomon H. M. Rhodehamel E. J. Kautter D. A. (1994). Growth and toxin production by *Clostridium botulinum* in sliced raw potatoes under vacuum with and without sulfite. J. Food Prot. 57, 878–881. doi: 10.4315/0362-028X-57.10.878, 31121695

[ref119] Stier R. F. Bell L. Ito K. A. Shafer B. D. Brown L. A. Seeger M. L. . (1981). Effect of modified atmosphere storage on *C. botulinum* toxigenesis and the spoilage microflora of salmon fillets. J. Food Sci. 46, 1639–1642. doi: 10.1111/j.1365-2621.1981.tb04450.x

[ref120] Sugiyama H. Woodburn M. Yang K. H. Movroydis C. (1981). Production of botulinum toxin in inoculated pack studies of foil-wrapped baked potatoes. J. Food Prot. 44, 896–899. doi: 10.4315/0362-028x-44.12.896, 30856729

[ref121] Tanaka N. (1982). Challenge of pasteurized process cheese spreads with *Clostridium botulinum* using in-process and post-process inoculation. J. Food Prot. 45, 1044–1050. doi: 10.4315/0362-028X-45.11.1044, 30913620

[ref122] Tanaka N. Goepfert J. M. Traisman E. Hoffbeck W. M. (1979). A challenge of pasteurized process cheese spread with *Clostridium botulinum* spores. J. Food Prot. 42, 787–789. doi: 10.4315/0362-028X-42.10.787, 30812122

[ref123] Tanaka N. Traisman E. Plantinga P. Finn L. Flom W. Meske L. . (1986). Evaluation of factors involved in antibotulinal properties of pasteurized process cheese spreads. J. Food Prot. 49, 526–531. doi: 10.4315/0362-028X-49.7.526, 30959639

[ref124] Ter Steeg P. F. Cuppers H. G. A. M. (1995). Growth of proteolytic *Clostridium botulinum* in process cheese products: II. Predictive modeling. J. Food Prot. 58, 1100–1108. doi: 10.4315/0362-028X-58.10.1100 31137382

[ref125] Ter Steeg P. F. Cuppers H. Hellemons J. C. Rijke G. (1995). Growth of proteolytic *Clostridium botulinum* in process cheese products: I. Data acquisition for modeling the influence of pH, sodium chloride, emulsifying salts, fat dry basis, and temperature. J. Food Prot. 58, 1091–1099. doi: 10.4315/0362-028X-58.10.1091, 31137370

[ref126] Torres-Baix E. Muñoz I. Gou P. Fulladosa E. Bover-Cid S. (2023). Computed tomography and predictive microbiology for non-invasive evaluation of the impact of dry-cured ham production process conditions on the behaviour of *Listeria monocytogenes* and *Clostridium botulinum*. Meat Sci. 202:109221. doi: 10.1016/j.meatsci.2023.109221, 37207553

[ref127] U.S. Dairy Export Council (2001). “Whey products, milk minerals and dairy calcium: new findings, benefits and applications,” in Applications Monograph: Calcium, ed. DiRienzo D. (Arlington, VA: U.S. Dairy Export Council).

[ref128] U.S. FDA CFSAN (United States Food and Drug Administration, Center for Food Safety and Applied Nutrition) (2000). Approximate pH of Foods and Food Products. Available online at: https://www.eidusa.com/Theory_pH_FOOD.htm (Accessed April 5, 2026).

[ref129] U.S. FDA CFSAN (United States Food and Drug Administration, Center for Food Safety and Applied Nutrition) (2007). Approximate pH of Foods and Food Products. Available online at: https://www.healthycanning.com/wp-content/uploads/pH-FDAapproximatepHoffoodslacf-phs.pdf (Accessed April 5, 2026).

[ref130] Vasconi M. Tirloni E. Stella S. Coppola C. Lopez A. Bellagamba F. . (2020). Comparison of chemical composition and safety issues in fish roe products: application of Chemometrics to chemical data. Foods 9:540. doi: 10.3390/foods9050540, 32349203 PMC7278670

[ref131] Vavrusova M. Liang R. Skibsted L. H. (2014). Thermodynamics of dissolution of calcium hydroxycarboxylates in water. J. Agric. Food Chem. 62, 5675–5681. doi: 10.1021/jf501453c, 24869479

[ref132] Virtanen P. Gommers R. Oliphant T. E. Haberland M. Reddy T. Cournapeau D. . (2020). SciPy 1.0: fundamental algorithms for scientific computing in Python. Nat. Methods 17, 261–272. doi: 10.1038/s41592-019-0686-2, 32015543 PMC7056644

[ref133] Wagenaar R. O. Dack G. M. (1958a). Factors influencing growth and toxin production in cheese inoculated with spores of *Clostridium botulinum* types a and B. I. Studies with surface-ripened cheese type I. J. Dairy Sci. 41, 1182–1190. doi: 10.3168/jds.S0022-0302(58)91072-5

[ref134] Wagenaar R. O. Dack G. M. (1958b). Factors influencing growth and toxin production in cheese inoculated with spores of *Clostridium botulinum* types a and B. II. Studies with surface-ripened cheese type II. J. Dairy Sci. 41, 1191–1195. doi: 10.3168/jds.S0022-0302(58)91073-7

[ref135] Wagenaar R. O. Dack G. M. (1958c). Factors influencing growth and toxin production in cheeseinoculated with spores of *Clostridium botulinum* types A and B. III. Studies with surface-ripened cheese type III. J. Dairy Sci. 41, 1196–1200. doi: 10.3168/jds.S0022-0302(58)91074-9

[ref136] Walstra P. Wouters J. T. M. Geurts T. J. (2005). Dairy Science and Technology, Second Edition. Boca Raton, FL: CRC Press, 31.

[ref137] Whiting R. C. Call J. E. (1993). Time of growth model for proteolytic *Clostridium botulinum*. Food Microbiol. 10, 295–301. doi: 10.1006/fmic.1993.1034

[ref138] Whiting R. C. Strobaugh T. P. (1998). Expansion of the time-to-turbidity model for proteolytic *Clostridium botulinum* to include spore numbers. Food Microbiol. 15, 449–453. doi: 10.1006/fmic.1998.0196

[ref139] Wood R. Cohen D. Munnelly K. (1975). Inhibitory effects of citrates in the determination of trace amounts of penicillin. Antimicrob. Agents Chemother. 8, 30–33. doi: 10.1128/AAC.8.1.30, 1164006 PMC429255

[ref140] Zhao L. Montville T. J. Schaffner D. W. (2002). Time-to-detection, percent-growth positive and maximum growth rate models for *Clostridium botulinum* 56A at multiple temperatures. Int. J. Food Microbiol. 77, 187–197. doi: 10.1016/s0168-1605(02)00111-3, 12160078

[ref141] Zybert A. Tarczyński K. Sieczkowska H. Krzęcio-Nieczyporuk E. Antosik K. (2020). Relationship of glycogen and lactate concentrations as a pork quality indicator. S. Afr. J. Anim. Sci. 50, 26–37. doi: 10.4314/sajas.v50i1.4

